# Causative role of PDLIM2 epigenetic repression in lung cancer and therapeutic resistance

**DOI:** 10.1038/s41467-019-13331-x

**Published:** 2019-11-22

**Authors:** Fan Sun, Liwen Li, Pengrong Yan, Jingjiao Zhou, Steven D. Shapiro, Gutian Xiao, Zhaoxia Qu

**Affiliations:** 1 UPMC Hillman Cancer Center, Pittsburgh, PA 15213 USA; 2 Department of Microbiology and Molecular Genetics, University of Pittsburgh School of Medicine, Pittsburgh, PA 15213 USA; 3 Department of Medicine, University of Pittsburgh Medical Center, University of Pittsburgh, Pittsburgh, PA 15261 USA; 4 Present Address: Chemical Biology Program, Memorial Sloan-Kettering Cancer Center, New York, NY USA

**Keywords:** Cancer therapeutic resistance, Lung cancer

## Abstract

Most cancers are resistant to anti-PD-1/PD-L1 and chemotherapy. Herein we identify PDLIM2 as a tumor suppressor particularly important for lung cancer therapeutic responses. While PDLIM2 is epigenetically repressed in human lung cancer, associating with therapeutic resistance and poor prognosis, its global or lung epithelial-specific deletion in mice causes increased lung cancer development, chemoresistance, and complete resistance to anti-PD-1 and epigenetic drugs. PDLIM2 epigenetic restoration or ectopic expression shows antitumor activity, and synergizes with anti-PD-1, notably, with chemotherapy for complete remission of most lung cancers. Mechanistically, through repressing NF-κB/RelA and STAT3, PDLIM2 increases expression of genes involved in antigen presentation and T-cell activation while repressing multidrug resistance genes and cancer-related genes, thereby rendering cancer cells vulnerable to immune attacks and therapies. We identify PDLIM2-independent PD-L1 induction by chemotherapeutic and epigenetic drugs as another mechanism for their synergy with anti-PD-1. These findings establish a rationale to use combination therapies for cancer treatment.

## Abstract (web summary)

PDLIM2 is repressed epigenetically in lung cancers, which are frequently resistant to anti-PD-1/PD-L1 and chemotherapy. Here, the authors describe the mechanism through which epigenetic restoration of PDLIM2 synergises with anti-PD-1 and chemotherapy in lung cancers.

## Introduction

Lung cancer is the leading cause of cancer-related deaths in both men and women and kills over 154,000 Americans each year^1^. Despite this statistic, the 5-year survival of lung cancer patients is only 19%, with minimal improvement in the past 30 years. Recent breakthrough in immunotherapies and in particular immune checkpoint PD-1/PD-L1 blockade for lung and several other cancers is inspiring; however, only a minority (about 20%) of patients benefit, with a response rate similar to that of chemotherapy^2–7^. Moreover, resistance may occur after an initial response. Currently, the mechanisms underlying the intrinsic and acquired resistance of lung cancer to chemo or PD-1/PD-L1 blockade therapy remain largely unknown. As a matter of fact, the mechanisms underlying lung cancer development and progression still remain largely unknown. The driver alternations have not yet been defined in about 50% of lung cancers, although mutations in several well-known oncogenes and tumor suppressor genes have been detected in certain lung cancers^8–10^. Improved understanding and better treatment options are direly needed for the No. 1 cancer killer globally and in the United States.

In vitro human cancer cell line studies suggested that the PDZ-LIM domain-containing protein PDLIM2, also known as SLIM or mystique^11–13^, may function as a tumor suppressor^14–20^. The main function of PDLIM2 is to promote ubiquitination and proteasomal degradation of nuclear activated NF-κB RelA and STAT3^21–23^, two master transcription factors that have been linked to lung and many other cancers^24–38^. However, the pathophysiological significance of these findings and in particular the role of PDLIM2 in lung cancer has not been studied. Of note, PDLIM2 is expressed highest in normal lungs^11–13^. Furthermore, whether PDLIM2 is involved in therapeutic resistance and whether PDLIM2 exerts its tumor suppressor role through targeting RelA and/or STAT3 have not been examined in any cancer type.

In this study, we employed a large panel of human lung tissue samples and cell lines as well as publically available big data to examine whether and how PDLIM2 is deregulated in human lung cancer and the pathogenic and clinical relevance of PDLIM2 deregulation. We also applied PDLIM2 global and lung epithelial-specific deletion mice, RelA lung epithelial-specific deletion mice, STAT3 lung epithelial-specific deletion mice, PDLIM2 and RelA or STAT3 double mutant mice, as well as three endogenous (spontaneous, and K-Ras oncogene and carcinogen urethane-induced) and two implanted (syngeneic and xenograft) lung cancer models to examine whether and how PDLIM2 is involved in lung cancer development and responses to anti-PD-1, chemo and epigenetic therapies. Moreover, we tested the different combination therapies of anti-PD-1, chemo and epigenetic drugs in our preclinical mouse models of lung cancer. These studies demonstrate PDLIM2 as a bona fide tumor suppressor that is particularly important for lung cancer therapeutic responses and importantly, can be targeted as a mono or combination therapy.

## Results

### Pathogenic and clinical significance of PDLIM2 in lung cancer

**Fig. 1:**
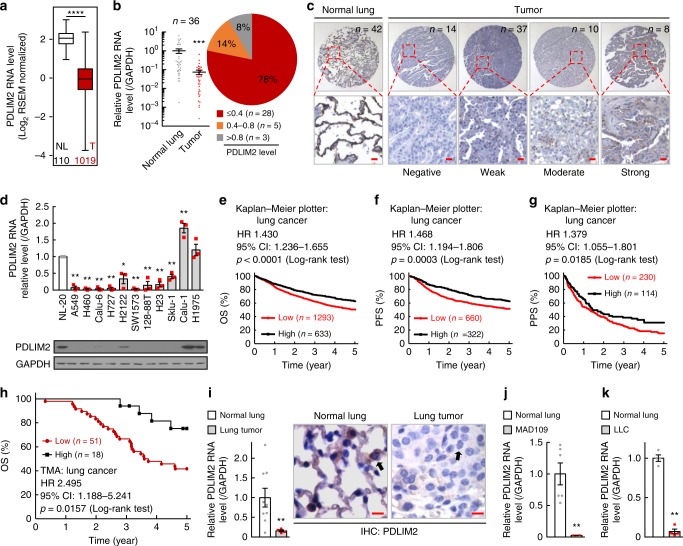
PDLIM2 is repressed in lung cancer, associating with poor prognosis. **a** TCGA data showing decreased PDLIM2 in human lung tumor (T, red column) compared to normal lung (NL). Sample numbers are indicated. The bottom-most and topmost horizontal lines, the lower and upper hinges, and the middle line of the boxplots indicate the minimum and maximum values, the 25th and 75th percentiles, and the median, respectively. **b** qPCR showing decreased PDLIM2 RNA expression in human lung tumors compared to normal lung tissues from the same patients. Percentiles of lung tumors with PDLIM2 RNA expression levels ≤0.4, between 0.4–0.8, or >0.8 of the average in normal lungs are also shown. **c** Lung tumor tissue microarray showing PDLIM2 repression in human lung cancer. Scale bar, 20 μm. **d** qPCR and immunoblotting (IB) showing decreased PDLIM2 in most human lung cancer cell lines (gray bars, *n* = 3). NL-20, normal human lung epithelial cell line (*n* = 3). **e**–**g** Kaplan–Meier survival curve generated from Kaplan–Meier Plotter showing positive association of PDLIM2 expression in tumors with overall survival (OS, **e**), progression free survival (PFS, **f**), and post progression survival (PPS, **g**) of lung cancer patients. **h** Kaplan–Meier survival curve on human lung tumor tissue microarray data in **c** showing positive association between PDLIM2 expression and patient survival. **i** qPCR and IHC staining analysis showing PDLIM2 repression in lung tumors induced by urethane in FVB/N mice (*n* ≥ 4). Representative lung epithelial and tumor cells are indicated by arrows. Scale bar, 10 μm. **j** qPCR analysis showing PDLIM2 repression in mouse lung cancer cell line MAD109 derived from a BALB/c mouse compared to normal lung tissues from BALB/c mice (*n* ≥ 4). **k** qPCR analysis showing PDLIM2 repression in mouse lung cancer cell line LLC derived from a C57BL/6 mouse, compared to normal lung tissues from C57BL/6 mice (*n* = 4). Student’s *t* test (two *t*ailed, unpaired) was performed in (**a**, **b**, **d**, **i**–**k**). Data represent means ± SEM in (**b**, **d**, **i**–**k**). *P < 0.05; **P < 0.01; ***P < 0.001; ****P < 0.0001.

To investigate the pathogenic and clinical significance of PDLIM2 in human lung cancer, we analyzed PDLIM2 expression in human lung cancers using The Cancer Genome Atlas (TCGA) database. Compared to normal lung tissues, the expression of PDLIM2 was significantly decreased in human lung cancers (Fig. 1a, Supplementary Fig. 1a, b, Supplementary Table 1,2). Analysis of the EMBL-EBI Expression Atlas database revealed that PDLIM2 was expressed at significantly low levels in 212 out of 287 human lung cancer cell lines (Supplementary Fig. 1c). Consistently, a significant decrease in PDLIM2 expression (<40% of the level in matched normal controls) was found in 28 out of 36 (based on the RNA level) and 51 out of 69 (based on the protein level) of our lung cancer samples directly isolated from patients (Fig. 1b, c, Supplementary Fig. 1d, Supplementary Table 3). PDLIM2 was also found to be significantly repressed at both RNA and protein levels in 9 out of 11 human lung cancer cell lines examined (Fig. 1d). More importantly, analysis of TCGA data and Kaplan–Meier Plotter, which combines TCGA, Gene Expression Omnibus (GEO, Affymetric microarrays only) and European Genome-phenome Archive (EGA) datasets, as well as our lung cancer tissue microarray assay and previously published lung cancer gene array data (www.ebi.ac.uk/arrayexpress; accession no.: E-MTAB-3665; Ref. ^25^) showed that PDLIM2 repression was associated with lung cancer progression, and poor overall survival (OS), progression free survival (PFS) and post progression survival (PPS) of patients (Fig. 1e–h, Supplementary Fig. 1e, f). Besides lung cancer, PDLIM2 was repressed in many other cancers (Supplementary Fig. 1g). PDLIM2 repression is thus clinically and pathogenically relevant to human cancers, particularly lung cancer.

Our mouse primary lung tumor and cell line studies indicated that PDLIM2 was also repressed in mouse lung cancer cells from different mouse models and mouse strains (Fig. 1i–k), suggesting that PDLIM2 repression is a common phenomenon of human and mouse lung cancers.

### Methylation and histone deacetylation of the *pdlim2* promoter in lung cancer

**Fig. 2:**
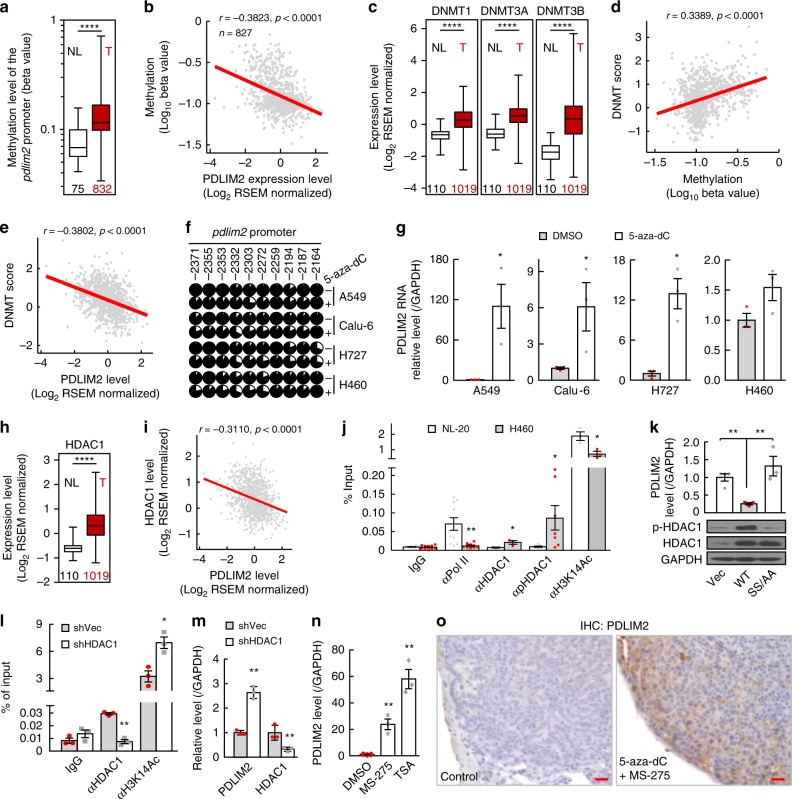
PDLIM2 repression in lung cancer involves promoter DNA methylation by DNMTs and promoter histone deacetylation by HDACs. **a**–**e** TCGA data showing increased *pdlim2* promoter methylation (**a**), negative association between PDLIM2 expression and its promoter methylation (**b**), increased DNMT expression (**c**), positive association between DNMT expression and *pdlim2* promoter methylation (**d**), and negative association between DNMT and PDLIM2 expression (**e**) in human lung cancer. T, tumors; NL, normal lungs. **f** Bisulfite genomic DNA sequencing showing 5-aza-dC de-methylation of the *pdlim2* promoter in the indicated human lung cancer cell lines. Each circle represents a CpG site. Ratio of the filled area represents the methylation percentile. The position of each CpG nucleotide relative to the PDLIM2 transcription initiation site (+1) is indicated at the top. **g** qPCR analysis showing PDLIM2 induction by 5-aza-dC in the indicated human lung cancer cell lines (*n* = 3). **h**, **i** TCGA data showing increased HDAC1 expression (**h**), and negative association between HDAC1 and PDLIM2 expression (**i**) in human lung cancer. **j** Chromatin immunoprecipitation (ChIP) showing increased HDAC1 and p-HDAC1 but decreased Pol II and H3K14Ac at the *pdlim2* promoter in H460 human lung cancer cells (*n* ≥ 3). **k** qPCR showing PDLIM2 down-regulation by WT but not SS/AA HDAC1 in H460 human lung cancer cells (*n* ≥ 3). **l** ChIP assay showing increased H3K14 acetylation at the *pdlim2* promoter by HDAC1 knockdown in H460 cells (*n* ≥ 3). **m**, **n** qPCR showing PDLIM2 up-regulation by HDAC1 knockdown or MS-275/TSA treatment in H460 cells (*n* ≥ 3). **o** IHC showing PDLIM2 recovery in mouse lung tumors by 5-aza-dC + MS-275. Scale bar, 20 μm. Sample numbers are indicated below the columns, and the bottom-most and topmost horizontal lines, the lower and upper hinges, and the middle line of the boxplots indicate the minimum and maximum values, the 25th and 75th percentiles, and the median, respectively, in (**a**, **c**, **h**). Student’s *t* test (two tailed, unpaired) was performed in (**a**, **c**, **g**, **h**, **j**-**n**). Data represent means ± SEM in (**g**, **j**–**n**). *P < 0.05; **P < 0.01; ***P < 0.001; ****P < 0.0001.

Our TCGA data analysis also revealed that the *pdlim2* promoter was hypermethylated in human lung cancers compared to normal lung tissues, and that the methylation of the *pdlim2* promoter was inversely associated with PDLIM2 expression (Fig. 2a, b, Supplementary Fig. 2a). Consistently, the expressions of all three functional DNA methyltransferases (DNMTs) were increased in human lung cancers, associating with *pdlim2* promoter methylation positively and PDLIM2 expression negatively (Fig. 2c–e). Interestingly, tobacco smoking, the most predominant risk factor that accounts for approximately 87% of lung cancers^39^, was associated positively with DNMT expression and *pdlim2* promoter methylation but negatively with PDLIM2 expression in human lung cancers (Supplementary Fig. 2b). More importantly, treatment with the DNMT inhibitor 5-aza-dC led to promoter hypomethylation and re-expression of PDLIM2 in human lung cancer cells (Fig. 2f, g).

In line with their synergistic role with DNMTs in suppressing tumor suppressor gene expression^40^, histone deacetylases (HDACs), particularly the class I members HDAC1 and HDAC2, were significantly increased in human lung cancers and inversely associated with PDLIM2 expression (Fig. 2h, i, Supplementary Fig. 2c–f). Accordingly, more HDAC1 and in particular its phosphorylation form (p-HDAC1), but less acetylated histone H3K14 (H3K14Ac) and RNA polymerase II (Pol II), were found at the *pdlim2* promoter in human lung cancer cells compared to normal lung epithelial cells (Fig. 2j). Ectopic expression of HDAC1 (WT), but not its phosphorylation deficient mutant (SS/AA), further suppressed PDLIM2 expression (Fig. 2k). Conversely, HDAC1 knockdown by shRNAs increased promoter-bound H3K14Ac and PDLIM2 transcription in human lung cancer cells (Fig. 2l, m). Also, HDAC2 knockdown increased PDLIM2 expression (Supplementary Fig. 2g). Treatment using the class I-specific HDAC inhibitor MS-275 or the pan HDAC inhibtor TSA induced PDLIM2 re-expression in human lung cancer cell lines or epithelial cell line transformed by K-Ras^Q61H^ oncogenic mutant (Fig. 2n, Supplementary Fig. 2h, i). Treatment using 5-aza-dC and MS-275 also restored PDLIM2 expression in murine lung cancers both in vitro and in vivo (Fig. 2o, Supplementary Fig. 2j). Thus, PDLIM2 repression in lung cancer involves its promoter methylation by DNMTs and de-acetylation by HDACs.

### Significance of PDLIM2 in lung cancer development

**Fig. 3:**
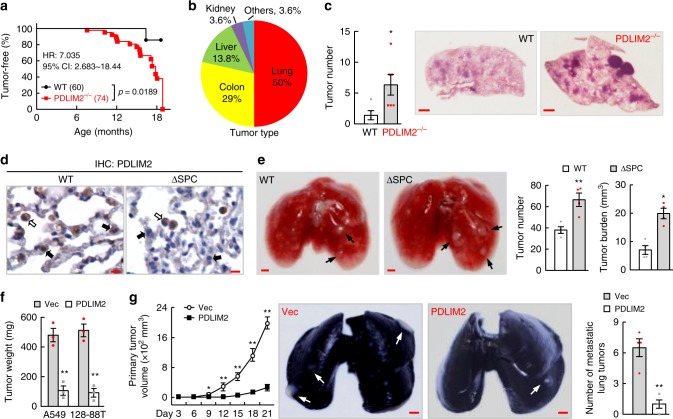
PDLIM2 loss increases tumorigenesis while its reconstitution suppresses lung cancer development and metastasis. **a** Kaplan–Meier tumor-free survival curve showing increased spontaneous tumors in PDLIM2^−/−^ mice compared to wild-type (WT) mice. Log-rank test was performed. **b** Percentile of tumor types spontaneously developed in PDLIM2^−/−^ mice showing a majority of lung tumors. **c** K-Ras^G12D^ model showing increased lung tumors in PDLIM2^−/−^ mice (WT: *n* = 5, PDLIM2^−/−^: *n* = 6). Scale bar, 1 mm. **d** Lung tissue IHC analysis showing specific deletion of PDLIM2 from lung epithelial cells in ΔSPC mice treated with urethane. Lung epithelial and myeloid cells are indicated by black and white arrows, respectively. Scale bar, 10 μm. **e** Urethane model showing increased lung tumors in ΔSPC mice (*n* = 4). Scale bar, 1 mm. **f** SCID mouse model showing lung tumor suppression by PDLIM2 reconstitution (*n* = 3). **g** MAD109 syngeneic model showing PDLIM2 suppression of primary and metastatic lung tumors (*n* = 4). Scale bar, 1 mm. Student’s *t* test (two tailed, unpaired) was performed, and data represent means ± SEM in (**c**, **e**–**g**). *P < 0.05; **P < 0.01.

To determine whether PDLIM2 epigenetic repression is just a bystander event or actually a driver of tumorigenesis, we examined whether PDLIM2 genetic deletion leads to development of spontaneous tumors, particularly lung tumor, in mice. Although PDLIM2-null mutant (PDLIM2^−/−^) mice appear normal and fertile^12^, notably, they started to develop spontaneous tumors at 7 months of age (Fig. 3a). By 19 months of age, all PDLIM2^−/−^ mice, compared to less than 10% of wild-type (WT) mice, spontaneously developed tumors. Of note, 50% of them were lung tumors (Fig. 3b, Supplementary Fig. 3a). PDLIM2^−/−^ mice were also more sensitive to K-Ras^G12D^-induced lung tumorigenesis, as evidenced by the significantly increased tumor number and tumor size (Fig. 3c).

To validate these findings, we created PDLIM2^flox/flox^/SP-C-rtTA^tg/−^/(tetO)7CMV-Cre^tg/tg^ (ΔSPC) mice, in which PDLIM2 can be selectively deleted from SP-C^+^ alveolar type II epithelial cells and bronchioalveolar stem cells, the main cells-of-origin of lung cancer^41–46^, after doxycycline administration (Fig. 3d). In comparison to WT mice, ΔSPC mice developed significantly more and larger lung tumors in response to urethane, a chemical carcinogen present in cigarette smoke, fermented food and alcoholic beverage (Fig. 3e, Supplementary Fig. 3b–d). Conversely, PDLIM2 reconstitution crippled in vitro the growth, migration and invasion ability and suppressed in vivo the tumor formation and metastasis of human and mouse lung cancer cell lines (Fig. 3f, g, Supplementary Fig. 3e–g). Altogether, these data demonstrated that PDLIM2 is a bona fide tumor suppressor that is particularly important for lung tumor suppression.

### Significance of PDLIM2 in lung cancer therapeutic responses

**Fig. 4:**
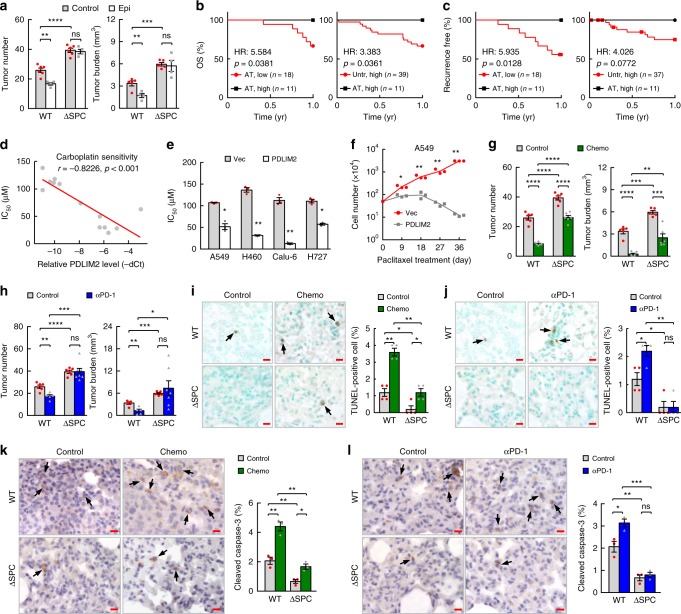
PDLIM2 is critical for lung cancer therapeutic responses. **a** Urethane model showing complete resistance to epigenetic therapy of lung cancer in ΔSPC mice (*n* ≥ 4). **b**, **c** Kaplan–Meier survival curve on human lung tumor data (GSE37745) showing better overall survival (OS, **b**) and recurrence-free survival (**c**) of patients with high PDLIM2 expression and received adjuvant treatment (AT), compared to that of patients with low PDLIM2 expression and received AT, or that of patients with high PDLIM2 expression but did not receive AT (Untr). Log-rank test was performed. **d**, **e** In vitro IC_50_ assay showing negative association between PDLIM2 expression and carboplatin sensitivity in lung cancer (**d**), and increased carboplatin sensitivity by PDLIM2 reconstitution (**e**) (*n* = 3). Pearson’s correlation test was performed in (**d**). **f** Cell growth assays showing increased paclitaxel sensitivity of A549 lung cancer cells by PDLIM2 reconstitution. Cells were treated with paclitaxel as shown in Supplementary Fig. 4a. **g** Urethane model showing increased carboplatin-paclitaxel chemo-resistance of lung cancer in ΔSPC mice (*n* ≥ 5). **h** Urethane model showing complete anti-PD-1 resistance of lung cancer in ΔSPC mice (*n* ≥ 5). **i**–**l** TUNEL assay (**i**,**j**) and IHC staining of cleaved caspase-3 (**k**, **l**) showing decrease in both basal and induced cancer cell apoptosis in ΔSPC mice (*n* ≥ 3). Scale bar, 10 μm. Student’s *t* test (two tailed, unpaired) was performed in (**a**, **e**–**l**). Data represent means ± SEM in (**a**, **e**, **g**–**l**). *P < 0.05; **P < 0.01; ***P < 0.001; ****P < 0.0001; ns, not statistically significant.

Since PDLIM2 expression can be restored by 5-aza-dC and MS-275, we examined whether these epigenetic drugs exert their antitumor activity via PDLIM2 restoration. In this regard, a lung cancer phase I/II trial study showed promising clinical benefits for their combination therapy, although the functional target genes remain largely unknown^47^. Consistent with the clinical study, 5-aza-dC and MS-275 combination (Epi) treatment showed promising therapeutic effect on lung tumor in WT mice, as evidenced by significant decrease in both tumor number and tumor burden (Fig. 4a). Remarkably, and somewhat unexpectedly, the same epigenetic treatment completely lost the ability to treat lung cancer in ΔSPC mice. Nevertheless, these data suggested that PDLIM2 restoration is essential for the anti-lung cancer activity of 5-aza-dC and MS-275.

To investigate whether PDLIM2 is involved in lung cancer therapeutic responses, we analyzed the 100 patients with known adjuvant treatment (AT) information (divided to 50/50 for PDLIM2 high/low, 96 of them with known information for recurrence-free survival) in the lung cancer cohort GSE37745^48^. Our analysis revealed that lung cancer patients with high PDLIM2 expression and received AT showed better overall survival and recurrence-free survival compared to those patients with low PDLIM2 expression and received AT, and those patients with high PDLIM2 expression but did not receive AT (Fig. 4b, c). In addition, PDLIM2 expression was inversely associated with the carboplatin sensitivity of lung cancer cells (Fig. 4d). These data suggested that PDLIM2 repression may contribute to lung cancer chemoresistance. Indeed, ectopic expression of PDLIM2 remarkably increased carboplatin sensitivity (Fig. 4e). PDLIM2 ectopic expression also overcame the acquired resistance to carboplatin and paclitaxel, another chemotherapeutic drug that is often used together with carboplatin as the first-line treatment for lung and many other cancers (Fig. 4f, Supplementary Fig. 4a–e). On the other hand, lung tumors in ΔSPC mice were more resistant to carboplatin-paclitaxel combination (Chemo) treatment compared to those in WT mice (Fig. 4g; Supplementary Fig. 4f).

Remarkably, lung tumors in ΔSPC mice were completely resistant to anti-PD-1 treatment, as evidenced by no change in either tumor number or tumor burden in ΔSPC mice but significant decrease in both tumor number and tumor burden in WT mice after anti-PD-1 treatment (Fig. 4h). Consistently, either basal apoptosis of lung cancer cells or those induced by chemotherapeutic drugs or anti-PD-1 were significantly lower in ΔSPC mice compared to that in WT mice (Fig. 4i–l). Of note, anti-PD-1 treatment failed to induce lung cancer cell death in ΔSPC mice. These data suggested that PDLIM2 epigenetic repression is one key mechanism underlying lung cancer resistance to both chemo- and anti-PD-1 therapies and in particular the PD-1 blockade therapy.

### Mechanisms underlying PDLIM2 inhibition of lung cancer and therapeutic resistance

It should be pointed out that the basal numbers of tumor-infiltrating T cells or those induced by chemo or anti-PD-1 treatment were comparable in ΔSPC and WT mice, although immune cells in mouse lungs showed complex responses (Supplementary Fig. 5). These data suggested that PDLIM2 repression may suppress tumor-infiltrating lymphocyte (TIL) activation and/or render lung cancer cells resistant to chemotherapeutic drugs and cytotoxic T cells (CTLs), including those CTLs released from the PD-1 checkpoint by PD-1 blockade.

**Fig. 5:**
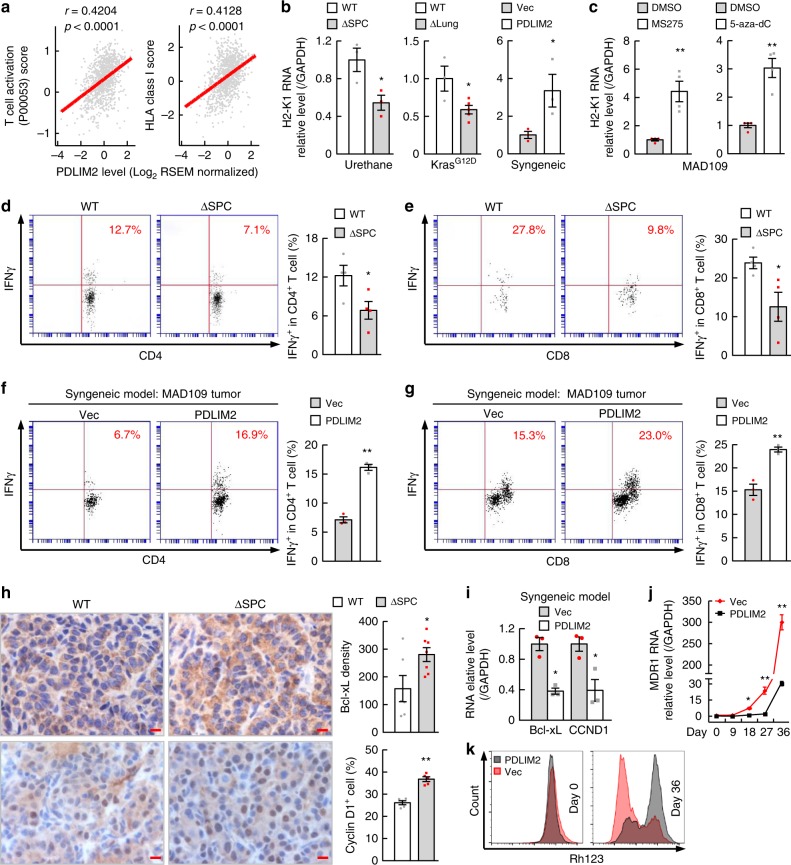
PDLIM2 increases MHC-I expression, represses MDR1 and cancer-related genes in lung cancer cells, and stimulates T-cell activation. **a** TCGA data showing positive association between PDLIM2 expression and T-cell activation or HLA Class I expression in human lung cancer. Pearson’s correlation test was performed. **b** qPCR showing down-regulation of the MHC-I H2-K1 expression by PDLIM2 deletion and up-regulation by PDLIM2 reconstitution in lung tumors (*n* ≥ 3). **c** qPCR showing up-regulation of H2-K1 expression in MAD109 lung cancer cells by epigenetic drugs (*n* = 4). **d**–**g** FACS analysis showing decreased lung T-cell activation in ΔSPC mice in urethane model (*n* = 4) and increased activation of tumor-infiltrating T cells by PDLIM2 reconstitution in MAD109 syngeneic model (*n* = 3). **h** IHC showing increased Bcl-xL and Cyclin D1 in lung tumors by PDLIM2 deletion (*n* ≥ 5). Scale bar, 10 μm. **i**, **j** qPCR showing PDLIM2 repression of Bcl-xL and Cyclin D1 (CCND1) expression in MAD109 tumors (**i**) and MDR1 induction in A549 lung cancer cells (**j**) (*n* = 3). **k** FACS of Rhodamine 123 (Rh123) showing PDLIM2 prevention of drug efflux from A549 cells. Student’s *t* test (two tailed, unpaired) was performed, and data represent means ± SEM in (**b**–**j**). *P < 0.05; **P < 0.01.

To this end, our analysis of the TCGA data indicated that expression of major histocompatibility complex class I (MHC-I) genes was decreased in human lung cancers, and that PDLIM2 expression was associated positively with T-cell activation and expression of MHC-I genes in human lung cancers but not in normal lung tissues (Fig. 5a, Supplementary Fig. 6a–c, Supplementary Tables 4, 5). Our in vivo mouse model studies showed that PDLIM2 deletion decreased while its expression increased MHC-I in lung cancer cells (Fig. 5b). In line with their ability in restoring PDLIM2 expression in lung cancer cells, epigenetic drugs 5-aza-dC and MS-275 could increase MHC-I expression in both mouse and human lung cancer cells (Fig. 5c, Supplementary Fig. 6d). Accordingly, PDLIM2 deletion from lung tumor cells diminished pulmonary T-cell activation in urethane mouse model of lung cancer, whereas re-expression of PDLIM2 in lung cancer cells increased tumor infiltration and activation of T cells and dendritic cells in syngeneic mouse model of lung cancer (Fig. 5d–g, Supplementary Fig. 6e–h). These data suggested that PDLIM2 is important for MHC-I expression, tumor antigen presentation and antitumor T-cell activation, thereby essential for the immune surveillance of lung tumor.

Our analysis of the TCGA data also revealed that PDLIM2 expression in human lung cancers was associated negatively with proliferation signature genes listed by Whitfield et al. in ref. ^49^ (Supplementary Fig. 7a, Supplementary Table 6). PDLIM2 deletion increased while its expression decreased expression of cell growth genes, including Bcl-xL and Cyclin D1 in lung cancer cells (Fig. 5h, i, Supplementary Fig. 7b, c). Consistently, PDLIM2 deletion decreased apoptosis and increased proliferation of lung cancer cells, whereas PDLIM2 re-expression showed opposite effects (Supplementary Fig. 7d, e). PDLIM2 repression also induces migration/invasion genes to promote lung tumor progression (Supplementary Fig. 7f–h). Taken together, these data suggested that PDLIM2 repression contributes to lung cancer immune evasion via repressing MHC-I expression/tumor antigen presentation and T-cell activation to evade CTLs on one hand, and inducing cell growth-related genes to resist CTL cytotoxicity on the other hand.

Obviously, survival gene induction by PDLIM2 repression also protects lung cancer cells from chemo-therapeutic drugs. Additional mechanism underlying lung cancer chemoresistance and in particular paclitaxel resistance by PDLIM2 repression involves induction of the multi-drug resistance gene MDR1. Whereas paclitaxel treatment induced MDR1 expression in lung cancer cells and subsequently drug efflux, PDLIM2 re-expression was sufficient to block paclitaxel induction of MDR1 and drug efflux (Fig. 5j, k, Supplementary Fig. 8).

**Fig. 6:**
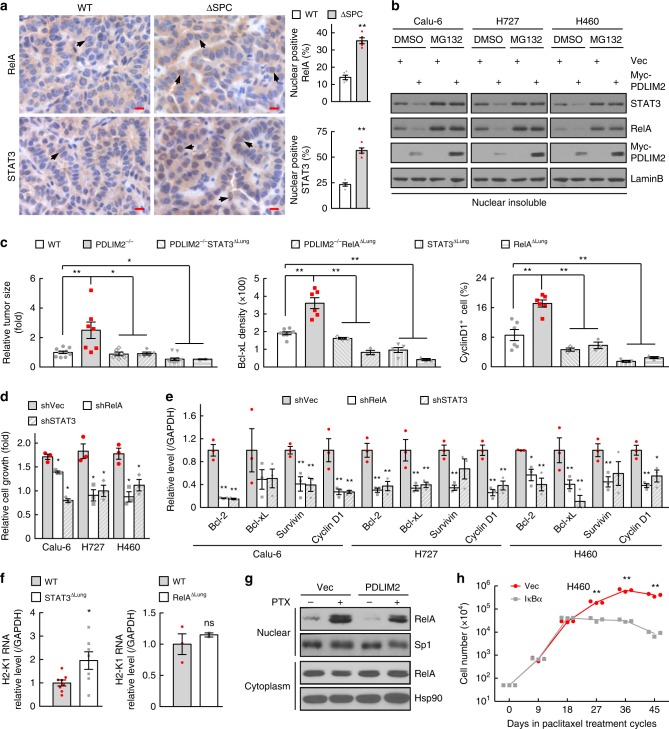
PDLIM2 represses STAT3 and RelA to increase MHC-I and repress cancer-related genes for suppressing lung cancer development and therapeutic resistance. **a** IHC staining showing increased nuclear RelA and STAT3 in lung cancers by PDLIM2 deletion in urethane model (*n* = 5). Scale bar, 10 μm. **b** Nuclear fraction-IB assays showing increased proteasomal degradation and decreased expression of nuclear RelA and STAT3 in the indicated human lung cancer cells reconstituted with PDLIM2 in the presence or absence of the proteasome inhibitor MG132. **c** K-Ras^G12D^ model showing suppression of increased lung tumors in PDLIM2^−/−^ mice by tumor-selective RelA or STAT3 deletion. Mouse numbers: WT, 10; PDLIM2^−/−^, 7; PDLIM2^−/−^STAT3^ΔLung^, 6; PDLIM2^−/−^RelA^ΔLung^, 5; STAT3^ΔLung^, 12; RelA^ΔLung^, 2. **d** Cell growth assays showing decreased growth of the indicated human lung cancer cells by RelA or STAT3 shRNA knockdown (KD) (*n* = 3). **e** qPCR analysis showing decreased expression of the indicated growth-related genes in the indicated human lung cancer cells by RelA or STAT3 KD (*n* = 3). **f** qPCR analysis showing increased H2-K1 expression in lung tumors by STAT3 deletion (*n* ≥ 7) but not RelA deletion (*n* ≥ 2) (urethane model). **g** Subcellular fraction-IB assays showing much lower paclitaxel induction of nuclear RelA in H460 human lung cancer cells reconstituted with PDLIM2. **h** Cell growth assays showing increased paclitaxel sensitivity of H460 cells by stable IκBα expression (*n* = 3). Student’s *t* test (two tailed, unpaired) was performed in (**a**, **c**–**f**, **h**). Data represent means ± SEM in (**a**, **c**–**f**). *P < 0.05; **P < 0.01; ns, not statistically significant.

We then examined whether PDLIM2 suppresses lung cancer through repressing the transcription factors NF-κB RelA and/or STAT3. Many genes identified above, particularly those involved in cell growth, migration and invasion, are known transcriptional targets of NF-κB and/or STAT3^50,51^. In line with its role in promoting ubiquitination and proteasomal degradation of nuclear RelA and STAT3^21–23^, PDLIM2 deletion increased while its expression decreased nuclear RelA and STAT3, a hallmark of NF-κB and STAT3 activation, in lung cancer cells (Fig. 6a, b). Notably, deletion of lung epithelial RelA or STAT3 blocked the increased lung tumorigenesis in PDLIM2^-/-^ mice (Fig. 6c). Consistently, RelA or STAT3 co-deletion or knockdown decreased growth gene expression and lung cancer cell growth (Fig. 6c–e, Supplementary Fig. 9). Interestingly, deletion of STAT3, but not RelA, increased MHC-I expression in lung cancer cells (Fig. 6f), indicating a specific role for STAT3. Conversely, PDLIM2 suppresses paclitaxel induction of MDR1 through RelA. Paclitaxel induced nuclear expression and binding to the *mdr1* promoter of RelA, in association with its functional partner NF-κB1 p50 (Fig. 6g, Supplementary Fig. 10a). PDLIM2 expression blocked all these effects of paclitaxel. In further support, blockage of RelA activation by another NF-κB inhibitor IκBα also overcame the paclitaxel resistance of lung cancer cells (Fig. 6h, Supplementary Fig. 10b). These data clearly indicated that PDLIM2 represses RelA and STAT3 to increase MHC-I expression and repress MDR1 and cancer-related genes, therefore suppressing lung cancer development and therapeutic resistance.

### Combinations of epigenetic agents, chemotherapeutic drugs, and anti-PD-1 for lung cancer treatment

**Fig. 7:**
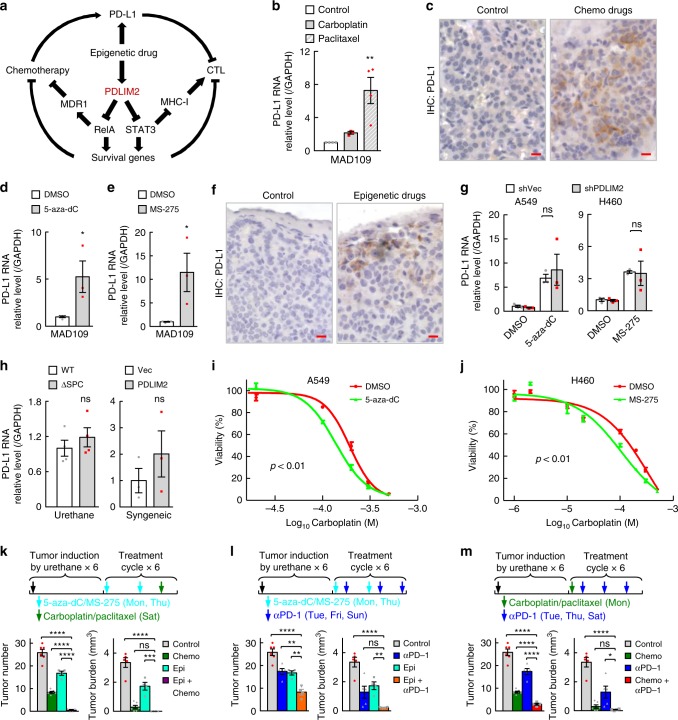
Epigenetic agents, chemotherapeutic drugs and anti-PD-1 synergize in lung cancer treatment. **a** Mechanistic rationale for combination therapy using anti-PD-1, chemotherapeutic drugs and epigenetic agents. **b** qPCR showing PD-L1 induction in lung cancer cells by carboplatin and paclitaxel (*n* ≥ 3). **c** IHC showing PD-L1 induction in lung tumors by chemotherapy in urethane model. Scale bar, 10 μm. **d**, **e** qPCR showing PD-L1 induction in lung cancer cells by 5-aza-dC or MS-275 (*n* ≥ 3). **f** IHC showing PD-L1 induction in lung tumors by epigenetic therapy in urethane model. Scale bar, 10 μm. **g** qPCR showing no significant effect of PDLIM2 knockdown on PD-L1 induction in lung cancer cells by epigenetic drugs (*n* = 3). **h** qPCR showing no significant effect on PD-L1 expression in lung tumors by PDLIM2 deletion (urethane model, *n* = 4) or by PDLIM2 reconstitution (MAD109 syngeneic mouse model, *n* = 3). **i**, **j** In vitro IC_50_ assay showing increased carboplatin sensitivity by 5-aza-dC in A549 cells and by MS-275 in H460 cells (*n* = 3). **k** Urethane model showing complete remission of almost all lung cancers by epigenetic and chemo combination therapy (*n* ≥ 4). **l**, **m** Urethane model showing better lung cancer treatment with epigenetic and anti-PD-1 combination therapy or chemo- and anti-PD-1 combination therapy (*n* ≥ 4). Student’s *t* test (two tailed, unpaired) was performed, and data represent means ± SEM in (**b**, **d**, **e**, **g**–**m**). *P < 0.05; **P < 0.01; ***P < 0.001; ****P < 0.0001; ns, not statistically significant.

Based on the findings above, we hypothesized that through restoring PDLIM2 expression to repress RelA and STAT3 activation, epigenetic drugs render lung cancer vulnerable to chemotherapeutic drugs and anti-PD-1 (Fig. 7a). We also hypothesized that PD-1/PD-L1 blockade overcomes the acquired immunoresistance induced by chemo and epigenetic therapies, because both our in vitro and in vivo studies showed that chemotherapeutic or epigenetic drugs induced PD-L1 on lung tumor cells and macrophages, although independently of PDLIM2 (Fig. 7a–h, Supplementary Fig. 11). Epigenetic agents indeed sensitized lung cancer cells to chemotherapeutic drugs in vitro (Fig. 7i, j). More importantly, their combination led to complete remission of almost all lung cancers in the mouse model (Fig. 7k). Epigenetic drugs and anti-PD-1 as well as anti-PD-1 and chemotherapeutic drugs also showed great synergies (Fig. 7l, m). These preclinical studies provide a strong rationale for use of combination therapy with anti-PD-1/PD-L1, chemotherapeutic and/or epigenetic drugs to treat lung cancer.

## Discussion

PD-1/PD-L1 blockade immunotherapy has recently joined chemotherapy as a standard treatment for lung cancer^6,7^. However, the majority of patients cannot benefit from these treatments, due to the intrinsic and acquired resistance of lung cancer cells^2–7^. Better understanding of lung cancer development and therapeutic resistance may form approaches to improve PD-1 blockade immunotherapy and chemotherapy and even lead to new treatments for the biggest cancer killer of both men and women. Through our human and mouse studies and analysis of the publicly available big data, we have demonstrated PDLIM2 as a bona fide tumor suppressor and its epigenetic repression and downstream STAT3/NF-κB RelA oncogenic activation as a main driver of lung cancer pathogenesis and resistance to anti-PD-1 and chemotherapeutic therapies that can be targeted as monotherapy or adjunctive therapy of anti-PD-1 and chemotherapeutic drugs.

While PDLIM2 is epigenetically repressed in more than 75% of all human lung cancer cases and PDLIM2 repression is associated with poor prognosis, global or lung epithelial-specific deletion of PDLIM2 in mice leads to increased lung cancer development and chemoresistance, and remarkably, complete anti-PD-1 resistance. Ectopic expression of PDLIM2 reverses the malignant phenotypes of lung cancer, and epigenetic agents, which can restore PDLIM2 expression, synergize with anti-PD-1 and notably with chemotherapeutic drugs for almost complete remission of lung cancer in the preclinical mouse model. Except for PDLIM2, epigenetic drugs also induce several other STAT3 and NF-κB negative regulators, such as SOCS3, PIAS3, and IκBα (Supplementary Fig. 12). Logically, these genes as well as many other target genes of epigenetic drugs, together with PDLIM2, decide the final outcome of the epigenetic treatment. Notably, specific deletion of lung cancer PDLIM2 completely nullifies the anti-lung cancer effect of epigenetic therapy, suggesting a pivotal role of PDLIM2 restoration in this potentially new lung cancer therapy. These findings also provide a new mechanistic insight into a phase I/II clinical trial study showing a promising response to epigenetic therapy in lung cancer patients^47^.

One main mechanism underlying PDLIM2 suppression of lung cancer development and therapeutic resistance involves repressing STAT3-dependent transcriptional repression of MHC-I and subsequent tumor immune evasion in the presence or absence of anti-PD-1 and chemotherapeutic drugs. It is noteworthy that STAT3 repression of MHC-I is dominant in lung cancer cells, although STAT1, another target of PDLIM2 for degradation, is known to induce MHC-I expression after activation by IFNγ^12,52^. In this regard, studies from us and others have shown that STAT3 is highly expressed and activated but STAT1 is frequently repressed in lung cancer^28,30,53^. More importantly, MHC-I, like PDLIM2, is repressed in lung cancer. Through repressing STAT3 and RelA, PDLIM2 also blocks the transcription of various cancer-related genes, such as MDR1 and those involved in cell survival, proliferation, migration and invasion, thereby suppressing tumor growth and progression and sensitizing tumor cells to the cytotoxicity of chemotherapeutic drugs as well as of the basal and induced CTLs by anti-PD-1 and chemotherapeutic drugs.

Interestingly, chemo and epigenetic drugs induce PDLIM2-independnet PD-L1 expression on lung cancer and associated macrophages, providing a different mechanism underlying lung cancer resistance to the conventional chemotherapy and potentially new epigenetic therapy. This finding also provides another mechanistic insight into our studies showing great synergies of anti-PD-1 with chemotherapeutic drugs and epigenetic agents.

Overall, our data demonstrate PDLIM2 epigenetic repression and RelA/STAT3 regulation of MHC-I, MDR1 and cancer-related genes as a previously unknown mechanism underlying lung cancer development and resistance to PD-1 blockade and chemotherapy. Our data also identify PDLIM2 restoration as a critical mechanism of epigenetic therapy and PDLIM2-independent PD-L1 induction as a new mechanism of acquired immune escape induced by chemo and epigenetic drugs. These data thus not only help understand lung cancer and therapeutic resistance, but also provide a firm basis for use of combination therapy with anti-PD-1/PD-L1, chemotherapeutic and/or epigenetic drugs to treat lung cancer. We believe that these knowledge and new combination therapies are applicable to many other cancer types, particularly given that PDLIM2 repression and RelA and STAT3 activation are common in human cancers.

## Methods

### Animals

We have complied with all relevant ethical regulations for animal testing and research. The animal experiments were performed in accordance with the US National Institutes of Health Guidelines on the Use of Laboratory Animals. All animals were maintained under pathogen-free conditions and used according to protocols approved by Institutional Animal Care and Use Committee of the University of Pittsburgh. PDLIM2^flx/flx^ mice were generated at the UC Davis Mouse Biology Program and possess loxP sites flanking exon 3 of the *pdlim2* gene and express a truncated PDLIM2 with 61 amino acids instead of 349 amino acids after Cre recombination. PDLIM2^−/−^ mice, RelA^flx/flx^ mice, SP-C-rtTA^tg/−^/(tetO)7CMV-Cre^tg/tg^ mice, STAT3^flx/flx^ mice and Lox-Stop-Lox (LSL) K-Ras^G12D^ mice have been described before^12,21–23,26,28,29,41,54–56^. BALB/c and C57BL/6 mice were originally from The Jackson Laboratory, and the severe combined immunodeficiency (SCID) mice were form Charles River.

### Lung cancer models

Spontaneous tumor model. PDLIM2^−/−^ BALB/c mice and control BALB/c mice were sacrificed at different ages for examining tumors in different organs/tissues.

Urethane-induced lung tumor model. 6–8-week-old mice under a pure FVB/N background were intraperitoneally (i.p.) injected with urethane (1 g/kg body weight, Sigma-Aldrich, St. Louis, MO, USA) once a week for six weeks. All mice were sacrificed for lung tumor examinations seven (for no drug treatment experiments) or six (for drug treatment experiments) weeks post urethane treatment. For drug treatment experiments, the epigenetic agents 5-aza-dC (1 mg/kg body weight, Sigma-Aldrich, St. Louis, MO, USA) and MS-275 (1 mg/kg body weight, Selleckchem, Houston, TX, USA), chemotherapeutic drugs carboplatin (30 mg/kg body weight, AdipoGen, San Diego, CA, USA) and paclitaxel (15 mg/kg body weight, AdipoGen, San Diego, CA, USA), and/or PD-1 neutralizing antibody (200 μg/mouse, BioXCell, West Lebanon, NH, USA) were i.p. injected as indicated in Fig. 7. Surface tumors in mouse lungs were counted by three blinded readers under a dissecting microscope, and tumor diameters were measured by microcalipers.

K-Ras^G12D^-induced lung tumor model. 7–10-week-old mice under mixed BALB/c background (F1 offsprings of C57BL/6 mice backcrossed to BALB/c mice 3 times) were intranasally administered 3 × 10^7^ plaque-forming units of Cre-expressing adenovirus (adenocre; Gene Transfer Vector Core, University of Iowa, Iowa City, IA). Nine weeks post AdenoCre treatment, all mice were sacrificed for lung tumor examinations.

MAD109 syngeneic lung tumor model. 6-week-old BALB/c mice were challenged subcutaneously (s.c.) in the right flank with 10^6^ MAD109 cells stably expressing ectopic PDLIM2 or the empty vector pQCXIP. Tumors at the injection sites were measured every three days and surgically taken out 21 days post MAD109 cell injection. All the mice were sacrificed 26 days post MAD109 cell injection and the lungs were perfused and stained with India ink (Speedball, NC, USA) for lung metastasis examinations.

LLC syngeneic lung tumor model. 6-week-old C57BL/6 mice were intravenously (i.v.) injected with 10^6^ LLC-Luc cells expressing ectopic PDLIM2 or an empty vector. All the mice were sacrificed 21 days post cell injection for lung metastasis examinations.

SCID mouse xenograft model. SCID mice were injected s.c. with 5 × 10^5^ the indicated human lung cancer cells expressing ectopic PDLIM2 or an empty vector. The recipient mice were sacrificed for tumor evaluation 14 days post injection.

### Cell lines and culture and treatment

The mouse lung cancer cell line MAD109 was obtained from Dr. Alan L. Epstein in Keck School of Medicine, University of Southern California in 2017. The mouse lung cancer cell line LLC-Luc and all the human lung cancer cell lines were obtained from colleagues in University of Pittsburgh between 2011 and 2015. The cell lines were grown in a humidified incubator (37 °C, 5% CO_2_), authenticated by short tandem repeat profiling, and tested for Mycoplasma (last tested in October 2018, IDEXX BioAnalytics). Culture medium were supplemented with 10% FBS. H23, H460, H727, H1975, H2122 cells were cultured in RPMI 1640. Calu-1 cells were cultured in McCoy’s 5a. Calu-6 and SK-LU-1 cells were cultured in EMEM. All the other cell lines were cultured in DMEM. The gene-expressing or knockdown stable cell lines were generated using the retroviral vector pQCXIP and the lentiviral vector pLL3.7, respectively. To avoid variations of different single cell clones, cell bulks after puromycin or GFP selection and validation of gene expression or knockdown were used for all the assays. For in vitro 5-aza-dC treatment experiment, cell culture medium was changed daily with fresh medium containing 5-aza-dC (0.5 μM) for 3 days. For in vitro MS-275 or TSA treatment experiment, cells were only treated for 24 h with drug concentration of 1.5 μM. For in vitro chemotherapeutic drug resistance assay, at day 3 of each cycle, drug treated-cells were washed with PBS and then cultured with normal medium. At day 5 and 7, cells were replenished with new culture medium. At day 9, cells were harvested, counted, and seeded for next cycle of the treatment.

### Flow cytometry (FACS) analysis

The cells were incubated with the antibodies against cell surface antigens after blocking with αCD16/CD32. The cells were then fixed with paraformaldehyde (2%), permeablized, and incubated with antibodies against intracellular antigens. For IFNγ staining, cells were treated with phorbol 12-myristate 13-acetate (PMA, 50 ng/ml), ionomycin (1 μM), brefeldin A (BFA, 3 μg/ml) and monensin (2 μM) for 4 h before they were stained for FACS analysis. Data were acquired and analyzed by Accuri C6 (BD Biosciences, Bedford, MA, USA) or analyzed using the FlowJo software^57,58^.

### Histology and immunohistochemistry (IHC)

Tissues were excised, fixed in formalin, embedded in paraffin, and cut into 5-μm-thick sections. The human lung tumor tissue microarrays (TMA) were described previously^26,29^. Sections were stained with H&E, or subjected to sequential incubations with the indicated primary antibodies, biotinylated secondary antibodies and streptavidin-HRP. The intensities of IHC staining were measured by ImageJ.

### In vivo BrdU labeling

Mice were i.p. injected with 50 mg/kg BrdU (Sigma-Aldrich, St. Louis, MO, USA) 24 h prior to sacrifice. Mouse lung tissue sections were stained with anti-BrdU (Sigma-Aldrich, St. Louis, MO, USA). More than 3000 cells per lung were counted in randomly selected fields. BrdU labeling index was calculated as the percentage of labeled cells per total cells counted.

### In vitro transwell migration and invasion assays

Cells were plated in the upper chamber of transwell coated with Matrigel (BD Biosciences, Bedford, MA, USA) (for invasion assay) or uncoated (for migration assay), and incubated for 24 h at 37 °C in 5% CO_2_. Nonmigrated cells were scraped from the upper surface of the membrane (8 μm pore size) with a cotton swab, and migrated cells remaining on the bottom surface were stained with crystal violet.

### Quantitative polymerase chain reaction (qPCR) analysis

The indicated tissues or cells were subjected to RNA extraction, RNA reverse transcription and real-time PCR using trizol, reverse transcriptase, and Power SYBR Green PCR Master Mix (Thermo Fisher Scientific, Waltham, MA USA) according to the product manufacture’s protocol^59,60^. Primer pairs used for qPCR were listed in Supplementary Table 8.

### Bisulfite genomic DNA sequencing

Genomic DNA from 5-aza-dC-treated or DMSO mock-treated cells were isolated using the PureLink Genomic DNA Purification Kit (Invitrogen, Carlsbad, CA, USA). Genomic DNA aliquots were then treated with sodium bisulfite using the EZ DNA Methylation-Gold Kit (Zymo Research, Irvine, CA, USA), followed by PCR to amplify the *pdlim2* promoter using Hot-Start Taq enzyme (Qiagen, Hilden, Germany). The PCR products were used for DNA sequencing to determine the methylation status of the CpG dinucleotides within the *pdlim2* promoter.

### Chromatin immunoprecipitation (ChIP) assays

Cells were collected after formaldehyde treatment. The chromatin DNA was extracted, broken into fragments of 300–1000 bp in length by sonication, and immunoprecipitated with antibodies to the target. IgG was used in immunoprecipitation as a control for nonspecific signal. DNA in the immunoprecipitation product was amplified by PCR. Antibodies used for histology, ChIP and FACS assays, including the company names, catalogue numbers, and dilutions, are listed in Supplementary Table 7. Primers for ChIP, qPCR and bisulfite genomic DNA amplification and sequencing are listed in Supplementary Table 8.

### Rhodamine 123 (Rh123) efflux assay

5 × 10^5^ cells treated with paclitaxel were suspended in 1 ml medium containing with 200 ng Rhodamine 123, and incubated at 37 °C for 1 h. Cells were then washed three times with PBS and re-suspended with Rh123-free medium for 90 min at 37 °C before being used for Rh123 detection by flow cytometry analysis.

### Subcellular fractionation and immunoblotting (IB) assays

Cytoplasmic, soluble, and insoluble nuclear extracts were prepared using the hypotonic buffer (20 mM HEPES, pH 8.0, 10 mM KCl, 1 mM MgCl_2_, 0.1% [vol/vol] Triton X-100, and 20% [vol/vol] glycerol), hypertonic buffer (20 mM HEPES, pH 8.0, 1 mM EDTA, 20% [vol/vol] glycerol, 0.1% [vol/vol] Triton X-100, and 400 mM NaCl), and insoluble buffer (20 mM Tris, pH 8.0, 150 mM NaCl, 1% [wt/vol] SDS, 1% [vol/vol] NP-40, and 10 mM iodoacetamide), respectively. The purity of the obtained fractions was confirmed by checking Hsp90 (cytoplasm), Sp1 (soluble nuclear fraction), or lamin B (insoluble nuclear fraction). Total nuclear extracts were prepared by simply lysing pellets in insoluble buffer after the cytoplasm was extracted. Whole-cell extracts were prepared by lysing cells in RIPA buffer (50 mM Tris-HCl, pH 7.4, 150 mM NaCl, 1 mM EDTA, 0.25% [wt/vol] Na-deoxycholate, 1% [vol/vol] NP-40, 1 mM DTT). All the lysis buffers were supplemented with 1 mM PMSF and a protease inhibitor cocktail (Roche Molecular Biochemicals). The cell extracts were used for IB assays^61–63^. Briefly, the cell extracts were separated on polyacrylamide gels followed by electrotransfer onto nitrocellulose membranes. After blocking nonspecific protein binding with 5% dry milk, the membranes were sequentially incubated with appropriate primary and horseradish peroxidase-conjugated secondary antibodies, with extensive wash with PBST after each of the incubation steps. Specific immune complexes were detected by enhanced chemiluminescence as specified by the manufacturer (Western Lightning ECL Pro; Amersham).

### Statistical analysis

Measurements were taken from distinct samples. Student’s *t* test (two tailed) was used to assess significance of differences between two groups. Log-rank test was used to compare overall patient survival between high and low PDLIM2 expression groups. Multivariate survival analysis was also performed using Cox’s proportional hazards model to statistically consider and adjust the potential effect of other clinical factors, such as age and tumor stage. Pearson’s correlation test was used to assess associations in expression between different genes. Except those for big data, all bars in figures represent means ± SEM. The *p* values are indicated as *p < 0.05, **p < 0.01, ***p < 0.001, ****p < 0.0001, ns, not statistically significant, except for those shown in figures. The *p* values < 0.05 and 0.01 are considered statistically significant and highly statistically significant, respectively.

### Reporting summary

Further information on research design is available in the Nature Research Reporting Summary linked to this article.

## Supplementary information

Supplementary Information

Reporting Summary

## Data Availability

The TCGA lung adenocarcinoma, lung squamous cell carcinoma, and lung cancer data we analyzed were obtained from https://portal.gdc.cancer.gov/projects. The dataset GSE37745 we analyzed was obtained from https://www.ncbi.nlm.nih.gov/geo/query/acc.cgi?acc=GSE37745. The authors declare that the main data supporting the findings of this study are available within the article and its Supplementary Information. Extra data that support the findings of this study are available from the corresponding authors upon reasonable request.

## Source data

Source Data
