# Supplementary material for: Causative role of PDLIM2 epigenetic repression in lung cancer and therapeutic resistance
Source: Nat Commun. 2019 Nov 22;10:5324. doi: 10.1038/s41467-019-13331-x (PMC6876573; doi:10.1038/s41467-019-13331-x)
Supplement: Supplementary file 1 — Supplementary Information [file 41467_2019_13331_MOESM1_ESM.pdf]

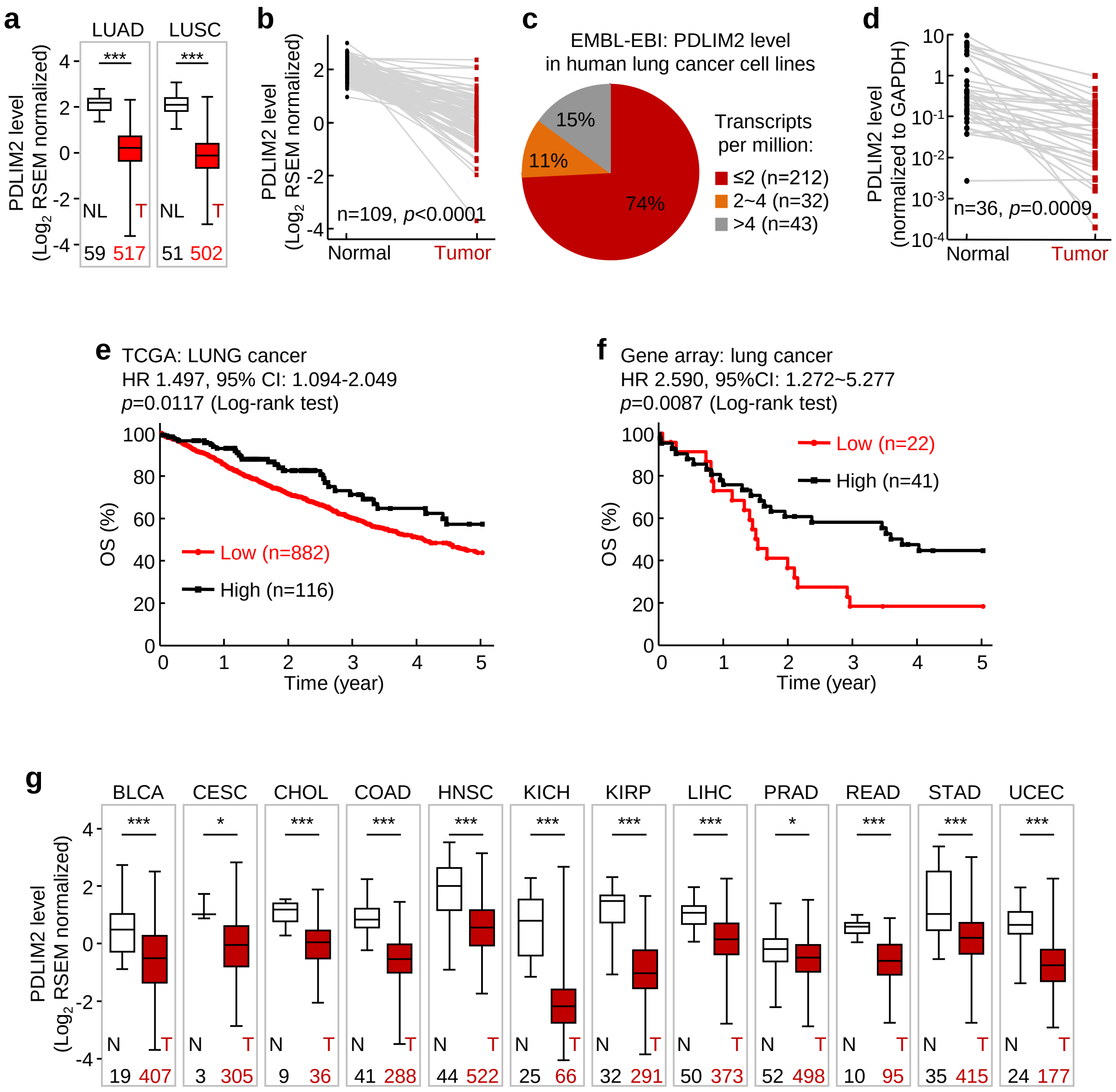


**Supplementary Fig. 1. PDLIM2 is repressed in lung cancer and other cancers, associating with poor prognosis**
**a** TCGA data showing decreased PDLIM2 expression in human lung adenocarcinoma (LUAD) and lung squamous cell carcinoma (LUSC; T: tumor; NL: normal lung). **b** TCGA data showing PDLIM2 repression in human lung cancer using paired lung cancer tissues and adjacent normal tissues from the same patients. **c** EMBL-EBI data showing PDLIM2 repression in human lung cancer cell lines. **d** qPCR analysis showing PDLIM2 repression in human lung cancer using paired lung cancer tissues and adjacent normal tissues from the same patients. **e**,**f** Kaplan-Meier survival curve of TCGA data (**e**) and gene array data (www.ebi.ac.uk/arrayexpress; accession no.: E-MTAB-3665) (**f**) respectively showing positive association between PDLIM2 expression and patient overall survival (OS). **g** TCGA data showing PDLIM2 repression in various human cancers besides lung cancer. BLCA, Bladder Carcinoma; CESC, Cervical Squamous Cell Carcinoma; CHOL, Cholangiocarcinoma; COAD, Colon Adenocarcinoma; HNSC, Head and Neck Squamous Cell Carcinoma; KICH, Kidney Chromophobe; KIRP, Kidney Renal Papillary Cell Carcinoma; LIHC, Liver Hepatocellular Carcinoma; PRAD, Prostate Adenocarcinoma; READ, Rectum Adenocarcinoma; STAD, Stomach Adenocarcinoma; UCEC, Uterine Corpus Endometrial Carcinoma. Student's *t* test (two tailed) was performed in (**a**), (**b**), (**d**), and (**g**), paired for (**b** and **d**), unpaired for (**a** and **g**). Sample numbers are indicated below the columns, and the bottom-most and topmost horizontal lines, the lower and upper hinges, and the middle line of the boxplots indicate the minimum and maximum values, the 25$^{th}$ and 75$^{th}$ percentiles, and the median, respectively, in (**a**) and (**g**). *P < 0.05; **P < 0.01; ***P < 0.001; ****P < 0.0001.

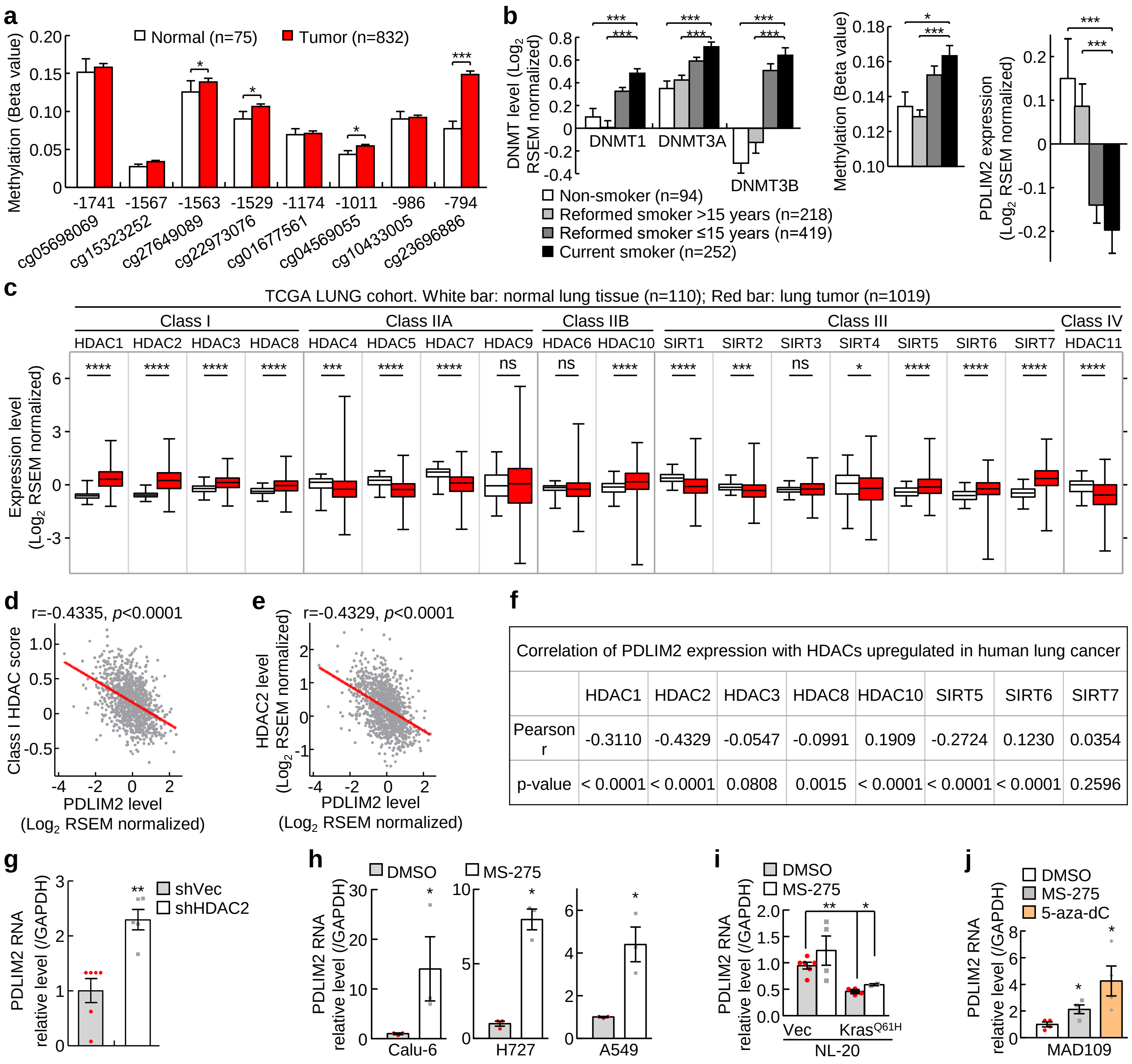


Correlation of PDLIM2 expression with HDACs upregulated in human lung cancer

| | HDAC1 | HDAC2 | HDAC3 | HDAC8 | HDAC10 | SIRT5 | SIRT6 | SIRT7 |
|---|---|---|---|---|---|---|---|---|
| Pearson r | -0.3110 | -0.4329 | -0.0547 | -0.0991 | 0.1909 | -0.2724 | 0.1230 | 0.0354 |
| p-value | < 0.0001 | < 0.0001 | 0.0808 | 0.0015 | < 0.0001 | < 0.0001 | < 0.0001 | 0.2596 |

**Supplementary Fig. 2. PDLIM2 repression in lung cancer involves promoter methylation by DNMTs and promoter histone deacetylation by HDACs**

**a** TCGA data showing increased CpG methylation of the *pdlim2* promoter in human lung cancer. The position of CpG nucleotides relative to the PDLIM2 transcription initiation site (+1) and their probes are indicated at the bottom. **b** TCGA data showing positive association between smoking and DNMT expression (left panel) or *pdlim2* promoter methylation (middle panel) and negative association between smoking and PDLIM2 expression (right panel) in human lung cancer. **c** TCGA data showing differential expressions of HDACs in human lung cancer. The bottom-most and topmost horizontal lines, the lower and upper hinges, and the middle line of the boxplots indicate the minimum and maximum values, the 25$^{th}$ and 75$^{th}$ percentiles, and the median, respectively. **d**,**e** TCGA data showing negative associations between PDLIM2 and class I HDACs (**d**) or HDAC2 (**e**) expression in human lung cancer. **f** TCGA data showing different correlations of PDLIM2 expression with various HDACs upregulated in human lung cancer. **g** qPCR analysis showing PDLIM2 up-regulation by HDAC2 knockdown in H460 lung cancer cells (n ≥ 5). **h-j** qPCR analysis showing PDLIM2 induction in the indicated human lung cancer cell lines by MS-275 (**h**), PDLIM2 repression by oncogenic K-Ras$^{Q61H}$ mutant and PDLIM2 re-induction by MS-275 in the normal human lung epithelial cell line NL-20 (**i**), and PDLIM2 re-induction by 5-aza-dC and MS-275 in mouse MAD109 lung cancer cells (**j**) (n ≥ 3). Student's *t* test (two tailed, unpaired) was performed in (**a-c** and **g-j**). Data represent means ± SEM in (**a-b** and **g-j**). *P < 0.05; **P < 0.01; ***P < 0.001; ****P < 0.0001. Pearson's correlation test was performed in (**d-f**).

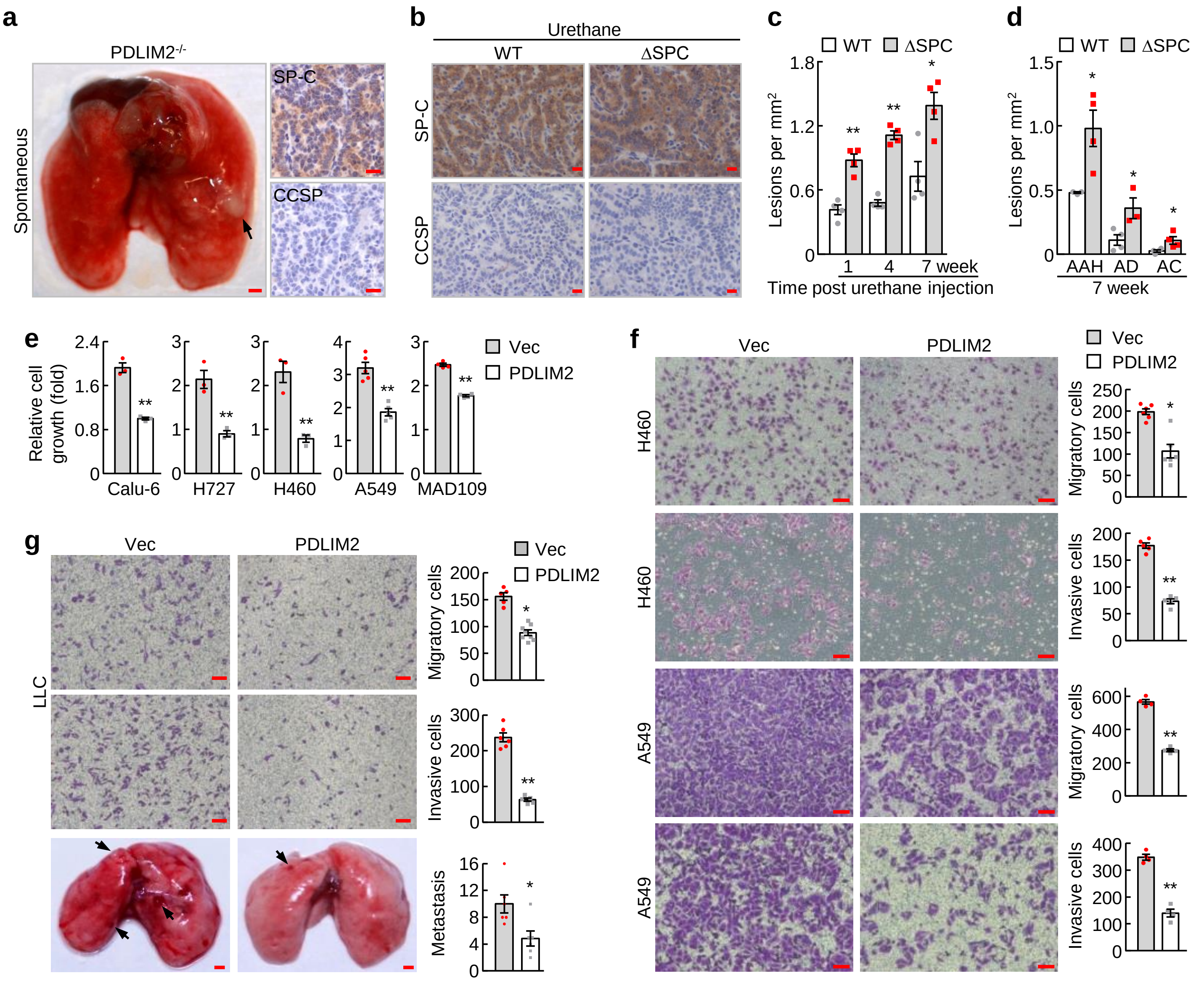


**Supplementary Fig. 3. Global or lung epithelial-specific deletion of PDLIM2 in mice leads to increased lung cancer development, while PDLIM2 re-constitution suppresses tumorigenicity of lung cancer cells both *in vitro* and *in vivo***

**a** Representative of lung tissues and IHC staining of SP-C and CCSP in spontaneous lung tumors from PDLIM2-null ($PDLIM2^{-/-}$) mice. Scale bar, 1 mm for whole lung picture, 20 μm for IHC staining. **b** Representative IHC analysis of SP-C and CCSP in lung tumors from urethane-treated WT and ΔSPC mice. Scale bar, 20 μm. **c** H&E staining showing increased lung lesions in ΔSPC mice at the indicated time points post urethane treatment (n = 4). **d** H&E staining showing increased atypical adenomatous hyperplasia (AAH), adenoma (AD) and adenocarcinoma (AC) in the lungs of ΔSPC mice 7 weeks post urethane treatment (n ≥ 3). **e** Cell growth assays showing decreased growth of the indicated human lung cancer cell lines and mouse lung cancer cell line MAD109 by PDLIM2 reconstitution (n ≥ 3). **f** *In vitro* migration and invasion assays showing decreased migration and invasion of the indicated human lung cancer cell lines by PDLIM2 reconstitution, respectively (n ≥ 4). Scale bar, 100 μm. **g** *In vitro* migration and invasion assays (n ≥ 5, Scale bar, 100 μm) and *in vivo* metastasis assays (n = 6, Scale bar, 1 mm) showing decreased migration, invasion and metastasis of the mouse lung cancer cell line LLC by PDLIM2 reconstitution. Student's *t* test (two tailed, unpaired) was performed, and data represent means ± SEM in (**c**-**g**). *P < 0.05; **P < 0.01.

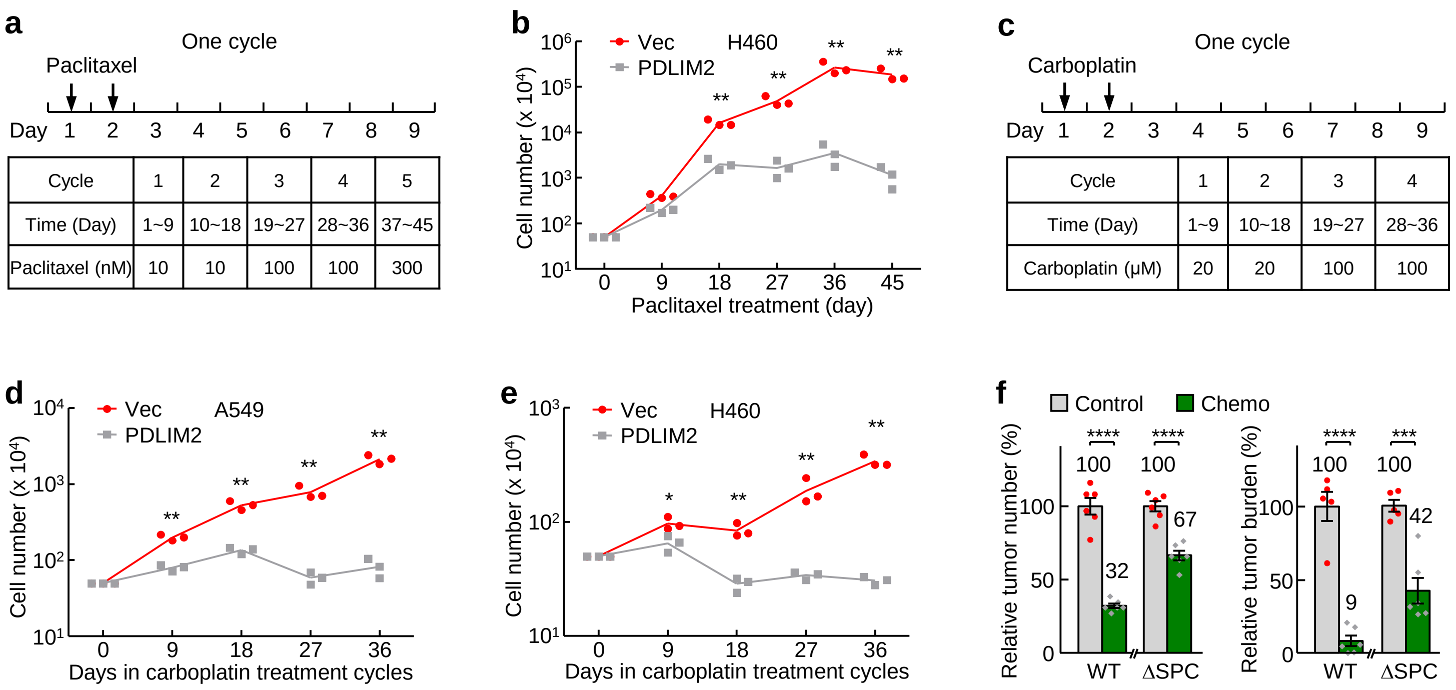


**Supplementary Fig. 4. PDLIM2 reconstitution renders lung cancer cells sensitive to chemotherapeutic drugs, while PDLIM2 selective deletion increases lung cancer chemo-resistance**

**a** Schedule of paclitaxel treatment of human lung cancer cells *in vitro*. **b** Cell growth assays showing increased paclitaxel sensitivity of H460 human lung cancer cells by PDLIM2 reconstitution. **c** Schedule of carboplatin treatment of human lung cancer cells *in vitro*. **d**, **e** Cell growth assays showing increased carboplatin sensitivity of A549 (**d**) and H460 (**e**) human lung cancer cells by PDLIM2 reconstitution. **f** Urethane model showing increased resistance to the carboplatin and paclitaxel combination therapy (Chemo) of lung cancer in ΔSPC mice (n ≥ 5). Student's *t* test (two tailed, unpaired) was performed, and data represent means ± SEM in (**b, d-f**). *P < 0.05; **P < 0.01; ***P < 0.001; ****P < 0.0001.

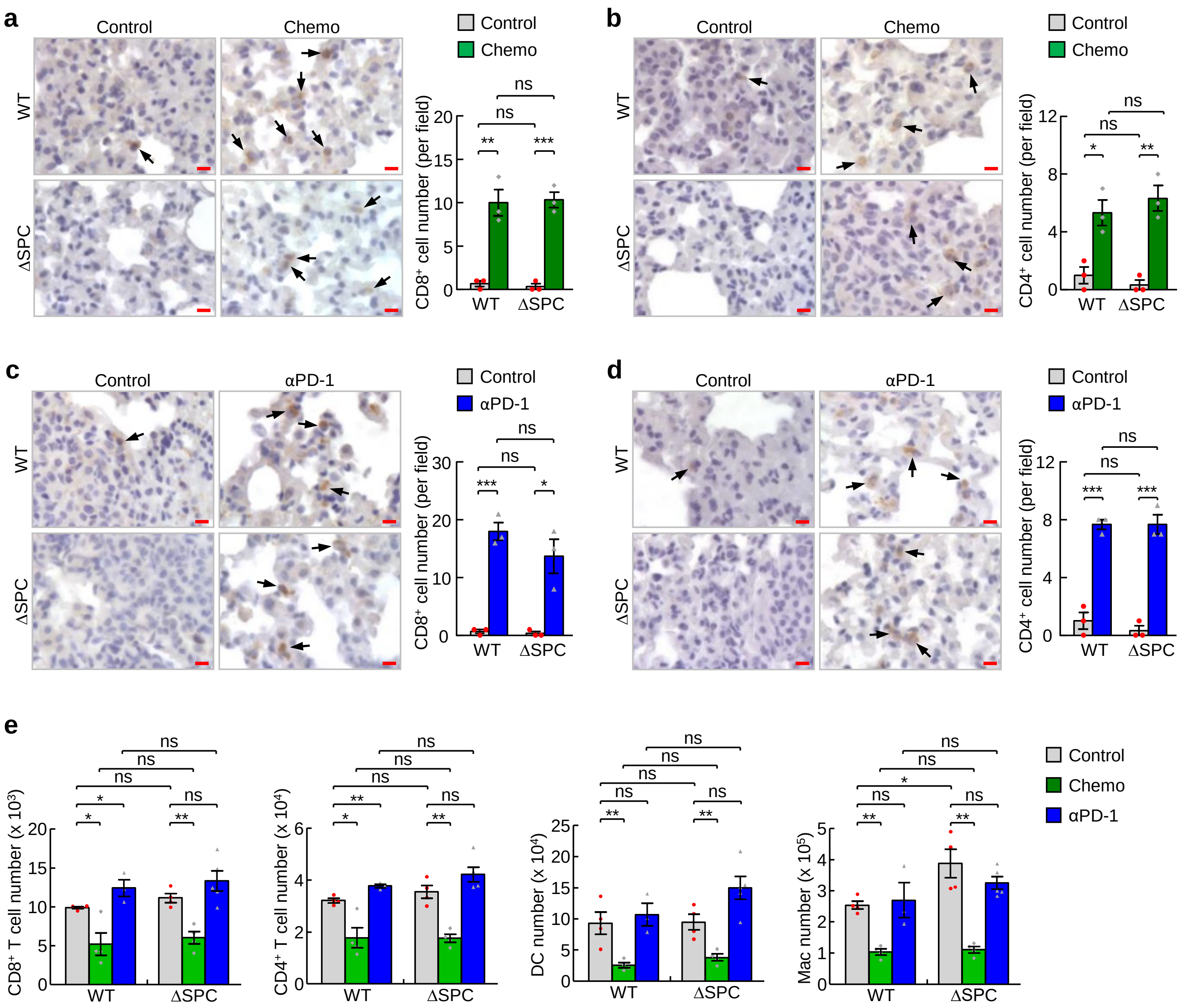


**Supplementary Fig. 5. PDLIM2 selective deletion does not affect the numbers of basal or treatment-induced tumor-infiltrating T cells, pulmonary T cells and dendritic cells**

**a** CD8 IHC staining of lung tumors from urethane-treated WT and ΔSPC mice with or without chemo-treatment (n = 3). Scale bar, 10 μm. **b** CD4 IHC staining of lung tumors from urethane-treated WT and ΔSPC mice with or without chemo-treatment (n = 3). Scale bar, 10 μm. **c** CD8 IHC staining of lung tumors from urethane-treated WT and ΔSPC mice with or without anti-PD-1 treatment (n = 3). Scale bar, 10 μm. **d** CD4 IHC staining of lung tumors from urethane-treated WT and ΔSPC mice with or without anti-PD-1 treatment (n = 3). Scale bar, 10 μm. **e**, FACS analysis showing cell numbers of the indicated immune cells in the lungs of WT and ΔSPC mice analyzed above (n = 3). Student's *t* test (two tailed, unpaired) was performed, and data represent means ± SEM in (**a**-**e**). *P < 0.05; **P < 0.01; ***P < 0.001; ****P < 0.0001; ns, not statistically significant.

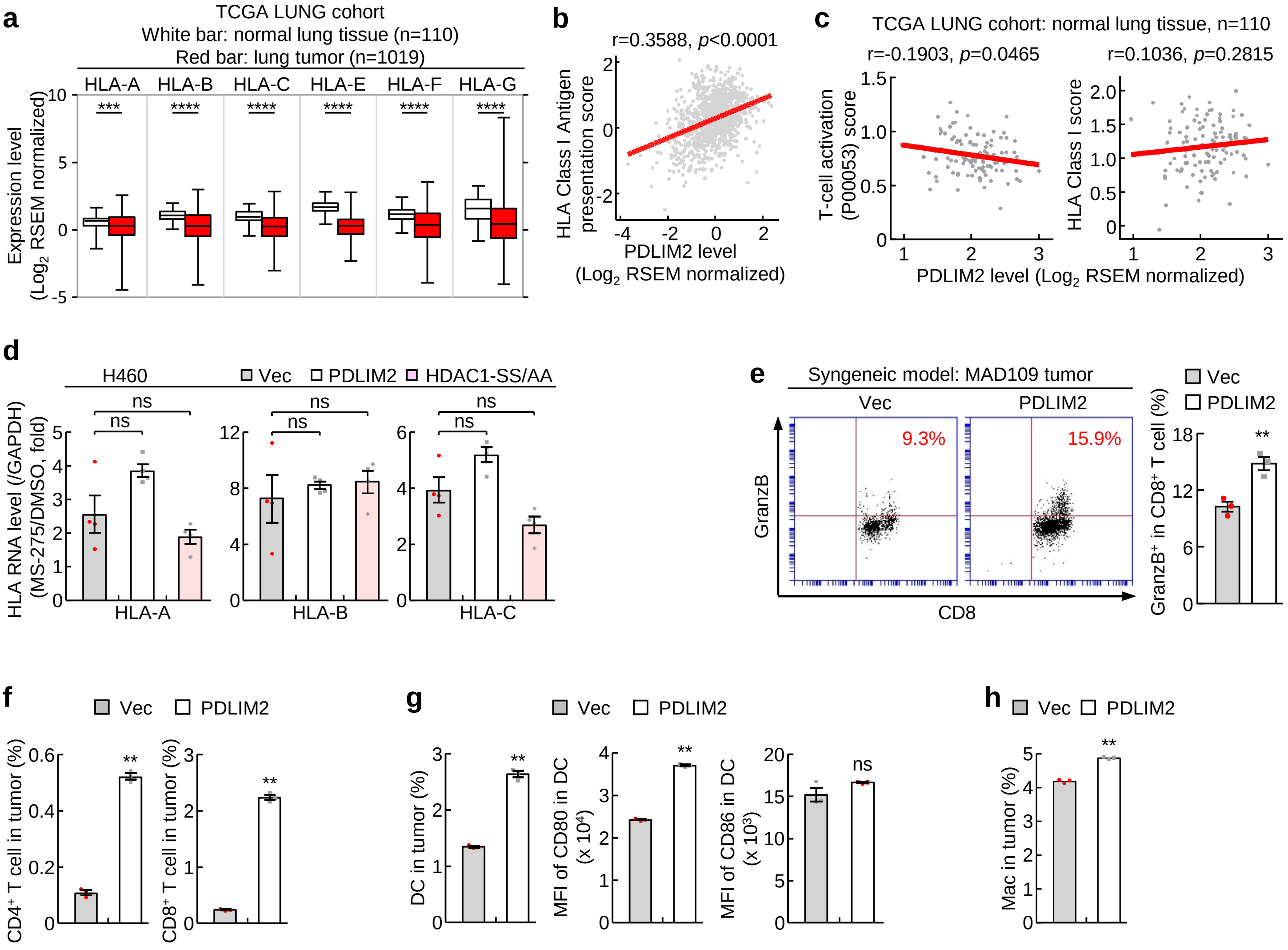


**Supplementary Fig. 6. PDLIM2 epigenetic repression is associated with HLA expression downregulation in human lung cancer and PDLIM2 expression in lung cancer cells stimulates T-cell activation and tumor infiltration**

**a** TCGA data showing decreased expression of MHC class I genes in human lung cancer. The bottom-most and topmost horizontal lines, the lower and upper hinges, and the middle line of the boxplots indicate the minimum and maximum values, the 25th and 75th percentiles, and the median, respectively. **b** TCGA data showing positive association between PDLIM2 expression and HLA class I antigen presentation-related genes in human lung cancer. **c** TCGA data showing negligible association between PDLIM2 expression and T-cell activation gene expression or expression of HLA class I genes in normal lung. **d** qPCR analysis showing similar induction of HLA genes by MS-275 in H460 human lung cancer cells stably expressing PDLIM2, HDAC1 mutant HDAC1-SS/AA or an empty vector (n = 4). **e** FACS analysis showing increased activation of tumor infiltrating $CD8^+$ T cells by PDLIM2 reconstitution in MAD109 syngeneic model (n = 3). **f** FACS analysis showing increased tumor infiltration of CD4 and CD8 T cells by PDLIM2 reconstitution in MAD109 syngeneic model (n = 3). **g** FACS analysis showing increased tumor infiltration and activation of dendritic cells (DC) by PDLIM2 reconstitution in MAD109 syngeneic model (n = 3). **h** FACS analysis showing increased tumor infiltration of macrophages (Mac) by PDLIM2 reconstitution in MAD109 syngeneic model (n = 3). Student's *t* test (two tailed, unpaired) was performed, and data represent means ± SEM in (**a** and **d**-**h**). *P < 0.05; **P < 0.01; ns, not statistically significant. Pearson's correlation test was performed in (**b**, **c**).

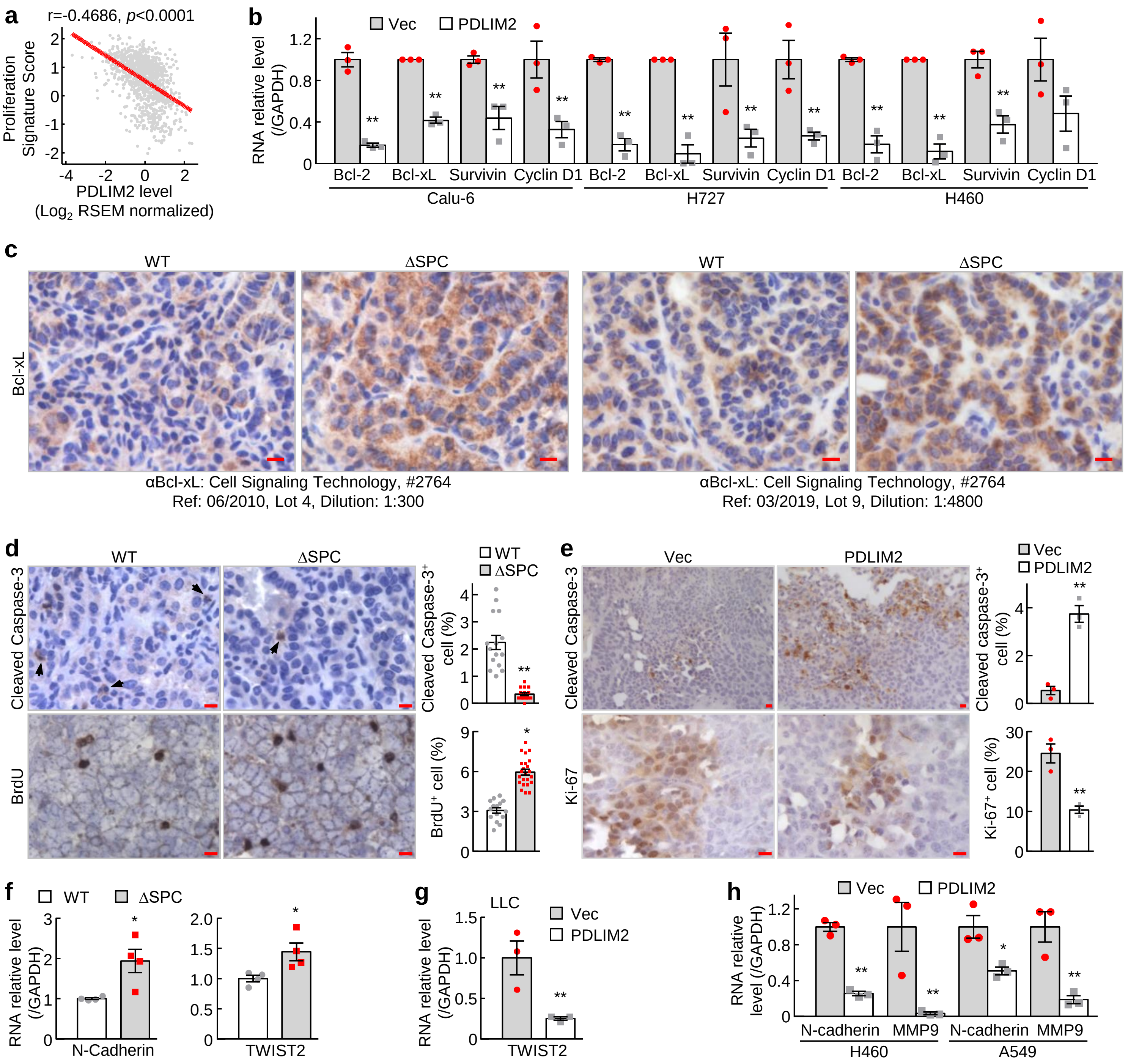


**Supplementary Fig. 7. PDLIM2 suppresses expression of cell growth, migration and invasion-related genes in lung cancer**

**a** TCGA data showing negative association between the expressions of PDLIM2 and proliferation signature genes in lung cancer. Pearson's correlation test was performed. **b** qPCR analysis showing decreased expression of Bcl-2, Bcl-xL, Survivin and Cyclin D1 in the indicated human lung cancer cell lines by PDLIM2 reconstitution (n = 3). **c** IHC showing increased Bcl-xL in lung tumors by PDLIM2 deletion. Scale bar, 10 μm. **d** IHC staining showing decreased apoptosis and increased proliferation of lung cancer cells in ΔSPC mice (urethane model) (n ≥ 15). Scale bar, 10 μm. **e** IHC staining showing increased apoptosis and decreased proliferation of lung cancer cells by PDLIM2 reconstitution in MAD109 syngeneic model (n = 3). Scale bar, 10 μm. **f** qPCR analysis showing increased N-Cadherin and TWIST2 expression in lung tumor cells from ΔSPC mice treated with urethane (n = 4). **g** qPCR analysis showing decreased TWIST2 expression in LLC mouse lung cancer cells reconstituted with PDLIM2 (n = 3). **h** qPCR analysis showing decreased N-Cadherin and MMP9 expression in the indicated human lung cancer cells reconstituted with PDLIM2 (n = 3). Student's *t* test (two tailed, unpaired) was performed, and data represent means ± SEM in (**b**, **d-h**). *P < 0.05; **P < 0.01.

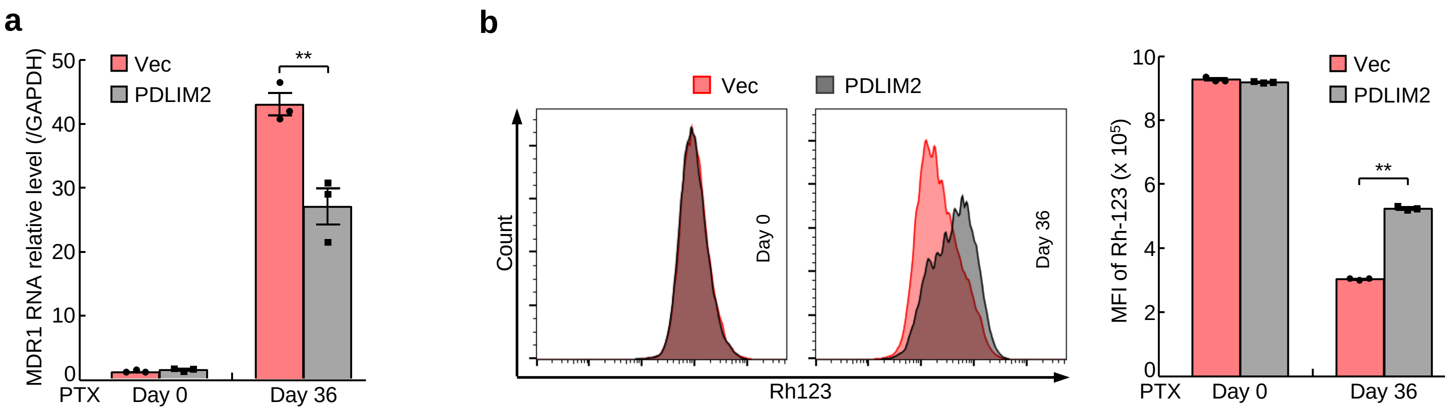


**Supplementary Fig. 8. PDLIM2 represses paclitaxel-induced MDR1 upregulation and drug efflux**
**a** qPCR showing PDLIM2 repression of MDR1 induction by paclitaxel (PTX) in H460 human lung cancer cells (n = 3). **b** FACS of Rhodamine 123 (Rh123) showing PDLIM2 suppression of drug efflux from H460 cells (n = 3). H460 cells were treated with paclitaxel as shown in Supplementary Fig. 4a. Student's *t* test (two tailed, unpaired) was performed, and data represent means ± SEM in (**a, b**). **P < 0.01.

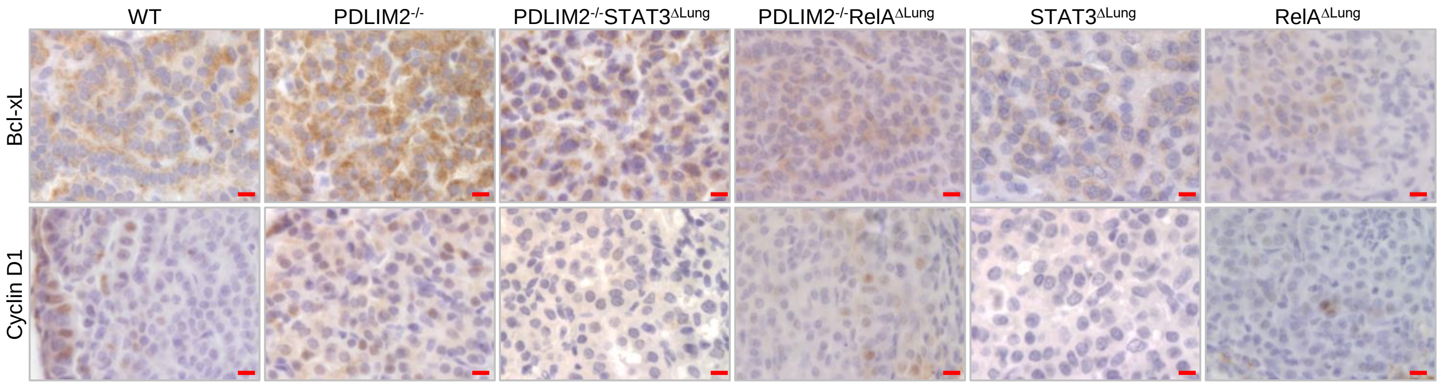


**Supplementary Fig. 9. RelA and STAT3 promote expression of cell growth-related genes in lung cancer cells**
IHC staining showing Bcl-xL and Cyclin D1 expression in lung tumors from the indicated mice (K-$Ras^{G12D}$ model). Scale bar, 10 μm.

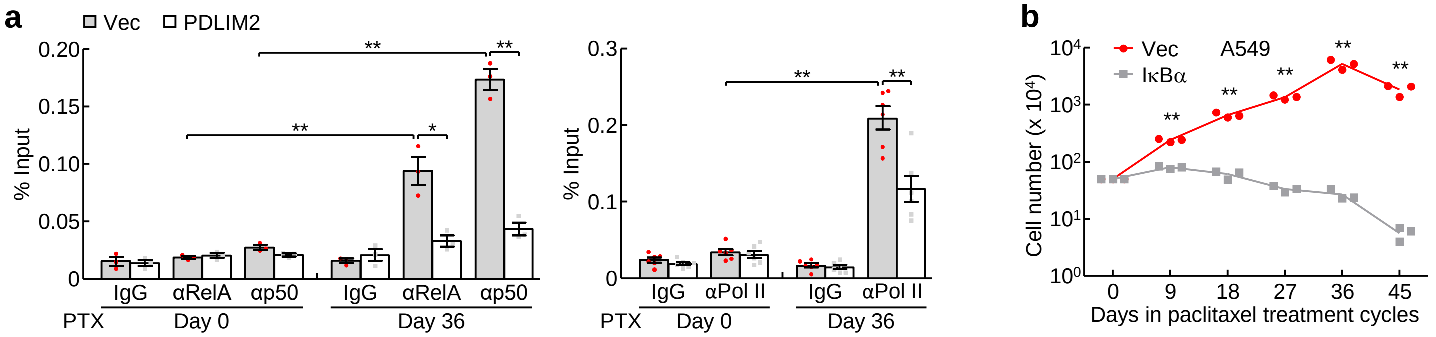


**Supplementary Fig. 10. PDLIM2 increases lung cancer cell sensitivity to paclitaxel through suppressing NF-κB-mediated MDR1 upregulation**

**a** ChIP assay showing PDLIM2 suppression of paclitaxel (PTX)-induced recruitment of NF-κB RelA/p50 and RNA polymerase II to the *mdr1* promoter in A549 lung cancer cells (n ≥ 3). **b** Cell growth assays showing increased paclitaxel sensitivity of A549 lung cancer cells by stable IκBα expression (n = 3). A549 cells with or without stable IκBα expression were treated with paclitaxel as shown in Supplementary Fig. 4a. Student's *t* test (two tailed, unpaired) was performed in (**a**, **b**), and data represent means ± SEM in (**a**). *P < 0.05; **P < 0.01.

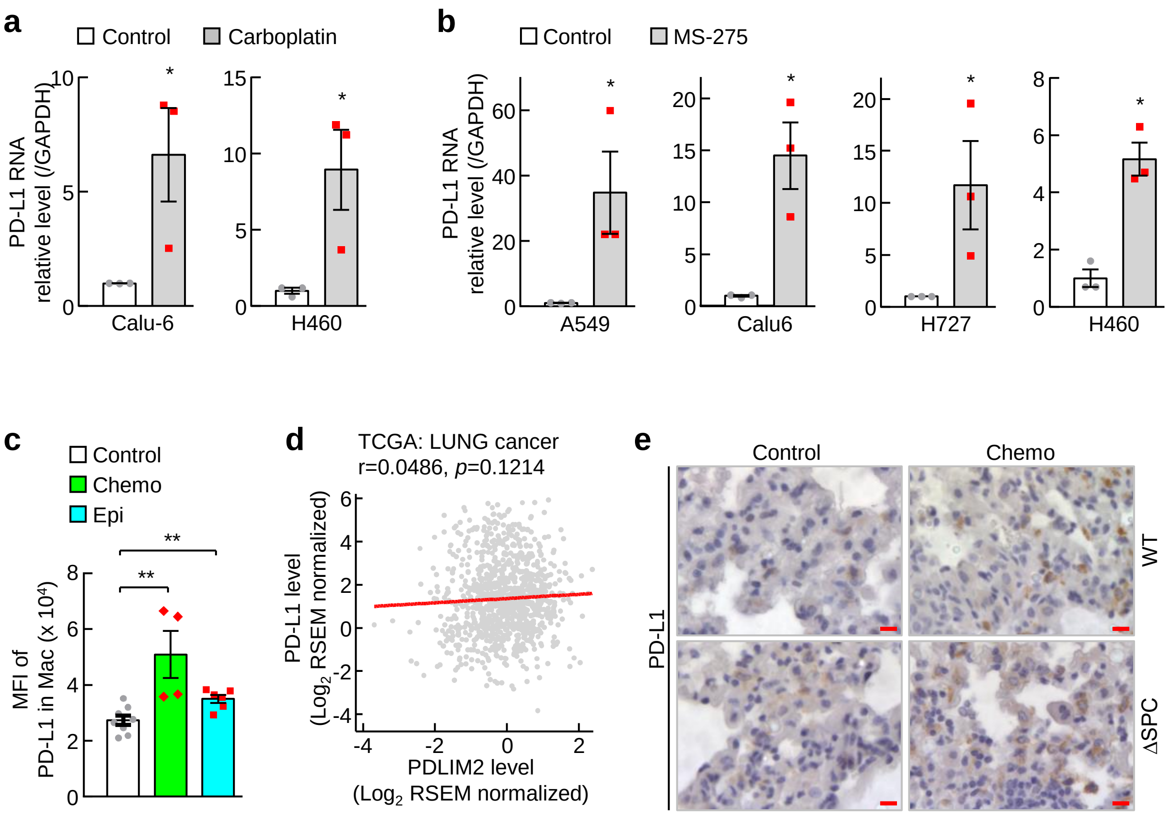


**Supplementary Fig. 11. Chemotherapeutic and epigenetic drugs induce PD-L1 expression in lung cancer cells and macrophages, which is PDLIM2-independent**

**a** qPCR showing PD-L1 induction in Calu-6 and H460 human lung cancer cells by carboplatin treatment (n = 3). **b** qPCR showing PD-L1 induction in the indicated human lung cancer cells by MS-275 treatment (n = 3). **c** FACS showing increased PD-L1 expression by chemo or epigenetic drugs in lung macrophages (Mac) of mice with lung tumors (urethane model) (n ≥ 4). **d** TCGA data showing no association between PDLIM2 and PD-L1 expression in human lung cancer. Pearson's correlation test was performed. **e** IHC staining showing similar PD-L1 increase in lung tumors of WT or ΔSPC mice by chemotherapy (urethane model). Scale bar, 10 μm. Student's *t* test (two tailed, unpaired) was performed, and data represent means ± SEM in (**a**-**c**). *P < 0.05; **P < 0.01.

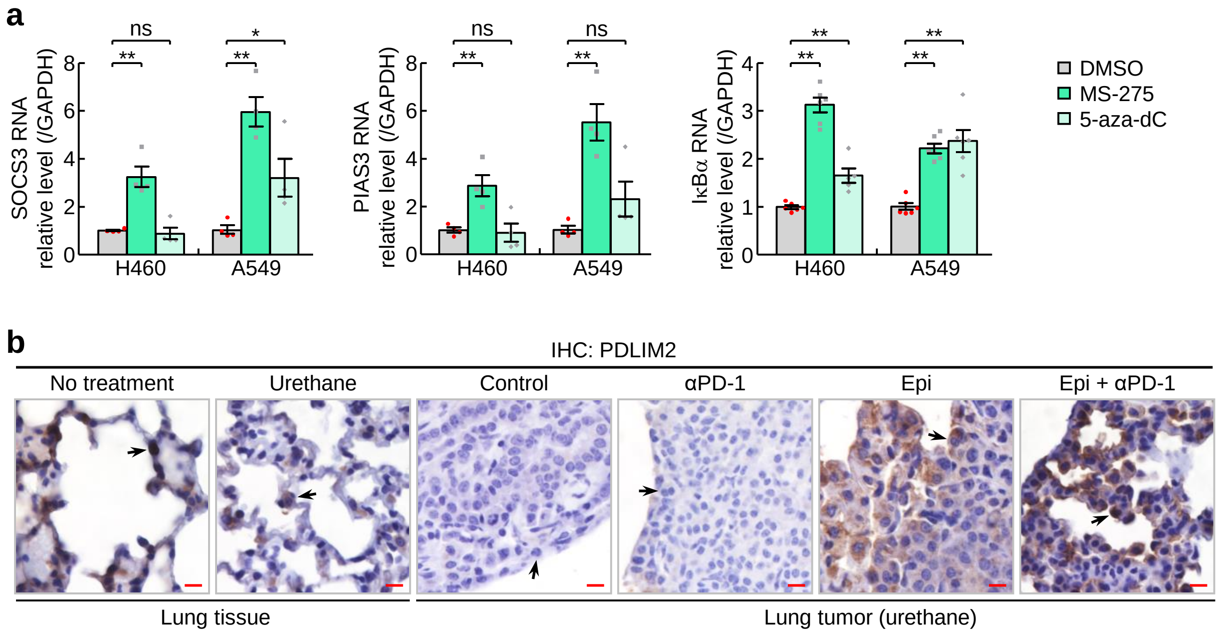


**Supplementary Fig. 12. The expression of STAT3 and RelA negative regulators is induced by epigenetic drugs in lung cancer cells and anti-PD-1 treatment has no effect on PDLIM2 expression or its recovery by epigenetic drugs in lung tumors**

(**a**) qPCR showing SOCS3, PIAS3, and IκBα induction in H460 and A549 human lung cancer cells by epigenetic drugs ($n \geq 4$). Student's *t* test (two tailed, unpaired) was performed, and data represent means ± SEM. *P < 0.05; **P < 0.01; ns, not statistically significant. (**b**) IHC staining showing no effect of anti-PD-1 treatment on PDLIM2 expression or its recovery by epigenetic drugs 5-aza-dC and MS-275 (Epi) in mouse lung tumors (urethane model). Scale bar, 10 μm.

**Supplementary Table 1. PDLIM2 expression and clinicopathological characteristics of lung cancer patients from TCGA LUNG cohort**

| **TCGA LUNG cohort** | **Expression level of PDLIM2** | | | |
|---|---|---|---|---|
| **Characteristics** | **Low (n=900)** | **High (n=119)** | **Total (n=1019)** | ***p*-value by $\chi^2$ test** |
| Sex | | | | 0.0002[a] |
| Male | 558 | 53 | 611 | |
| Female | 341 | 66 | 407 | |
| N/A | 1 | 0 | 1 | |
| Age (mean=66.28yrs) | | | | 0.8107 |
| < mean | 410 | 52 | 462 | |
| > mean | 466 | 62 | 528 | |
| N/A | 24 | 5 | 29 | |
| Smoke | | | | 0.0144[b] |
| Non-smoker | 76 | 18 | 94 | |
| Reformed smoker (>15yrs) | 192 | 26 | 218 | |
| Reformed smoker (≤15yrs) | 375 | 44 | 419 | |
| Current smoker | 227 | 25 | 252 | |
| N/A | 30 | 6 | 36 | |
| T stage | | | | 0.0002[c] |
| T1 | 234 | 50 | 284 | |
| T2 | 521 | 50 | 571 | |
| T3 | 103 | 15 | 118 | |
| T4 | 39 | 3 | 42 | |
| N/A | 3 | 1 | 4 | |
| N stage | | | | 0.0371[d] |
| N0 | 567 | 85 | 652 | |
| N1 | 204 | 23 | 227 | |
| N2 | 107 | 7 | 114 | |
| N3 | 7 | 0 | 7 | |
| N/A | 15 | 4 | 19 | |
| Clinical stage | | | | 0.0239[e] |
| I | 452 | 73 | 525 | |
| II | 256 | 30 | 286 | |
| III | 161 | 12 | 173 | |
| IV | 29 | 4 | 33 | |
| N/A | 2 | 0 | 2 | |
| Recurrence | | | | 0.9221 |
| Yes | 263 | 37 | 300 | |
| No | 501 | 69 | 570 | |
| N/A | 136 | 13 | 149 | |
| Metastasis[f] | | | | 0.0304 |
| Yes | 371 | 38 | 409 | |
| No | 345 | 57 | 402 | |
| N/A | 184 | 24 | 208 | |

[a]: More PDLIM2-high patients in female is due to less smoke (Supplementary Table 2). [b]: Non-smoker *vs* Reformed smoker (>15yrs, ≤15yrs) and Current smoker. [c]: T1 *vs* T2, T3, and T4. [d]: N0 *vs* N1, N2, and N3. [e]: I *vs* II, III, and IV. [f]: Metastasis was determined by M stage, N stage, and new tumor event with distant metastasis.

**Supplementary Table 2. Sex and smoke history of lung cancer patients from TCGA LUNG cohort**

| TCGA LUNG Cohort | | | | |
|---|---|---|---|---|
| **Characteristic** | **Male (n=611)** | **Female (n=407)** | **Total (n=1018)** | ***p*-value by $\chi^2$ test** |
| Smoke | | | | <0.0001 |
| Non-smoker | 31 | 63 | 94 | |
| Reformed smoker (>15yrs) | 140 | 78 | 218 | |
| Reformed smoker (≤15yrs) | 243 | 176 | 419 | |
| Current smoker | 173 | 79 | 252 | |
| N/A | 24 | 11 | 35 | |

**Supplementary Table 3. PDLIM2 expression and clinicopathological characteristics of lung cancer patients from tissue microarray (TMA)**

| TMA | Expression level of PDLIM2 | | | |
|---|---|---|---|---|
| **Characteristics** | **Low (n=51)** | **High (n=18)** | **Total (n=69)** | ***p*-value by $\chi^2$ test** |
| Race | | | | 0.2860 |
| White | 44 | 17 | 61 | |
| Black | 3 | 0 | 3 | |
| N/A | 4 | 1 | 5 | |
| Sex | | | | 0.6859 |
| Female | 25 | 10 | 35 | |
| Male | 25 | 8 | 33 | |
| N/A | 1 | 0 | 1 | |
| Pleural | | | | 0.0889 |
| Yes | 26 | 5 | 31 | |
| No | 25 | 13 | 38 | |
| Inflammation | | | | 0.0422[a] |
| Mild | 41 | 18 | 59 | |
| Moderate | 7 | 0 | 7 | |
| Severe | 3 | 0 | 3 | |
| T stage | | | | 0.0199[b] |
| T1 | 15 | 11 | 26 | |
| T2 | 28 | 7 | 35 | |
| T3 | 1 | 0 | 1 | |
| T4 | 6 | 0 | 6 | |
| N/A | 1 | 0 | 1 | |
| N stage | | | | 0.1579[c] |
| N0 | 30 | 14 | 44 | |
| N1 | 9 | 2 | 11 | |
| N2 | 8 | 1 | 9 | |
| NX | 3 | 1 | 4 | |
| N/A | 1 | 0 | 1 | |
| Clinical stage | | | | 0.0485[d] |
| 1/1a/1b | 26 | 14 | 40 | |
| 2 | 7 | 2 | 9 | |
| 3a/3b | 14 | 1 | 15 | |
| N/A | 4 | 1 | 5 | |
| Recurrence | | | | 0.1071 |
| Yes | 25 | 5 | 30 | |
| No | 23 | 12 | 35 | |
| N/A | 3 | 1 | 4 | |
| Metastasis[e] | | | | 0.0361 |
| Yes | 31 | 6 | 37 | |
| No | 17 | 11 | 28 | |
| N/A | 3 | 1 | 4 | |

[a]: Mild *vs* Moderate and Severe. [b]: T1 *vs* T2, T3 and T4. [c]: N0 *vs* N1 and N2. [d]: 1/1a/1b *vs* 2 and 3a/3b. [e]: Metastasis was determined by M stage, N stage, and new tumor event with distant metastasis.

**Supplementary Table 4. Positive association between PDLIM2 expression and T-cell activation-related genes expression (P00053) in lung cancer by TCGA data analysis.** Only those genes with r > 0.3 are listed.

| Gene | Pearson r | *p*-value |
|---|---|---|
| B2M | 0.3409 | < 0.0001 |
| CD247 | 0.3046 | < 0.0001 |
| CD28 | 0.4021 | < 0.0001 |
| CD3E | 0.3089 | < 0.0001 |
| CD74 | 0.4859 | < 0.0001 |
| CD80 | 0.3716 | < 0.0001 |
| CD86 | 0.3791 | < 0.0001 |
| GRAP2 | 0.3225 | < 0.0001 |
| HLA-DMA | 0.4589 | < 0.0001 |
| HLA-DMB | 0.4150 | < 0.0001 |
| HLA-DOA | 0.4222 | < 0.0001 |
| HLA-DPA1 | 0.4629 | < 0.0001 |
| HLA-DQA1 | 0.3774 | < 0.0001 |
| HLA-DQA2 | 0.3552 | < 0.0001 |
| HLA-DRA | 0.4501 | < 0.0001 |
| LAT | 0.3257 | < 0.0001 |
| LCP2 | 0.3904 | < 0.0001 |
| NFATC1 | 0.3020 | < 0.0001 |
| NFATC2 | 0.3326 | < 0.0001 |
| PIK3CD | 0.3903 | < 0.0001 |
| PIK3CG | 0.3515 | < 0.0001 |
| PPP3CC | 0.4455 | < 0.0001 |
| PTPRC | 0.3684 | < 0.0001 |
| RAC2 | 0.4019 | < 0.0001 |
| VAV1 | 0.3748 | < 0.0001 |
| WAS | 0.4732 | < 0.0001 |
| ZAP70 | 0.3217 | < 0.0001 |

**Supplementary Table 5. Positive association of PDLIM2 expression with HLA class I antigen presentation-related genes expression in lung cancer by TCGA data analysis**

| Gene | Pearson r | *p*-value |
|---|---|---|
| HLA-A | 0.3336 | < 0.0001 |
| HLA-B | 0.3678 | < 0.0001 |
| HLA-C | 0.3738 | < 0.0001 |
| HLA-E | 0.4285 | < 0.0001 |
| HLA-F | 0.4096 | < 0.0001 |
| HLA-G | 0.3027 | < 0.0001 |
| B2M | 0.3409 | < 0.0001 |
| TAP1 | 0.1062 | 0.0007 |
| TAP2 | 0.1573 | < 0.0001 |
| TAPBP | 0.2714 | < 0.0001 |
| CALR | -0.0160 | 0.6092 |
| CANX | 0.0060 | 0.8479 |
| PDIA3 | -0.0722 | 0.0213 |

**Supplementary Table 6. Negative association of PDLIM2 expression with proliferation signature genes expression in lung cancer by TCGA data analysis**

| Gene | Pearson r | *p*-value |
|---|---|---|
| BIRC5 | -0.4083 | < 0.0001 |
| AURKB | -0.4230 | < 0.0001 |
| CDC6 | -0.4452 | < 0.0001 |
| CKS2 | -0.3668 | < 0.0001 |
| TRAIP | -0.3938 | < 0.0001 |
| CHEK1 | -0.3916 | < 0.0001 |
| PTTG1 | -0.3995 | < 0.0001 |
| DNMT1 | -0.2457 | < 0.0001 |
| NASP | -0.2751 | < 0.0001 |
| UNG | -0.4462 | < 0.0001 |
| CDC7 | -0.4068 | < 0.0001 |
| FEN1 | -0.4308 | < 0.0001 |
| MCM3 | -0.3890 | < 0.0001 |
| MCM4 | -0.4199 | < 0.0001 |
| MCM5 | -0.2167 | < 0.0001 |
| MCM6 | -0.4047 | < 0.0001 |
| ORC1L | -0.4328 | < 0.0001 |
| PCNA | -0.3767 | < 0.0001 |
| PRIM1 | -0.3726 | < 0.0001 |
| RFC1 | -0.2287 | < 0.0001 |
| RRM1 | -0.3732 | < 0.0001 |
| RRM2 | -0.4002 | < 0.0001 |
| TOP2A | -0.4817 | < 0.0001 |
| MAD2L1 | -0.4326 | < 0.0001 |
| CENPE | -0.4278 | < 0.0001 |
| BUB1 | -0.4556 | < 0.0001 |
| CTPS | -0.3553 | < 0.0001 |
| DHFR | -0.2562 | < 0.0001 |
| TYMS | -0.3987 | < 0.0001 |
| CCNA2 | -0.4214 | < 0.0001 |
| CCNB1 | -0.4358 | < 0.0001 |
| CCNE1 | -0.2881 | < 0.0001 |
| CCNF | -0.3187 | < 0.0001 |
| CDC20 | -0.3899 | < 0.0001 |
| DDX11 | -0.2521 | < 0.0001 |
| E2F3 | -0.2801 | < 0.0001 |
| MKI67 | -0.3803 | < 0.0001 |
| PKMYT1 | -0.2369 | < 0.0001 |
| PLK1 | -0.3805 | < 0.0001 |
| TIMP1 | 0.3417 | < 0.0001 |
| CDC25C | -0.4607 | < 0.0001 |
| CENPF | -0.4693 | < 0.0001 |
| MAPK13 | -0.0335 | 0.2852 |
| EXOSC9 | -0.1879 | < 0.0001 |
| MYB | -0.1930 | < 0.0001 |

## Supplementary Table 7. Antibodies Used

| Antibody | Company | Cat. No. | Dose | Usage |
|---|---|---|---|---|
| Anti-Cleaved Caspase 3 | Cell Signaling Technology, Danvers, MA, USA | 9661 | 1:200 | IHC |
| Anti-Bcl-xL | Cell Signaling Technology, Danvers, MA, USA | 2764 | 1:300 | IHC |
| Anti-PD-L1 | Cell Signaling Technology, Danvers, MA, USA | 64988 | 1:200 | IHC |
| Anti-BrdU | Sigma-Aldrich, St. Louis, MO, USA | B2531 | 1:500 | IHC |
| Anti-Cyclin D1 | Santa Cruz Biotechnology, Dallas, TX, USA | sc-450 | 1:200 | IHC |
| Anti-Ki-67 | Santa Cruz Biotechnology, Dallas, TX, USA | sc-7846 | 1:500 | IHC |
| Anti-SP-C | Santa Cruz Biotechnology, Dallas, TX, USA | sc-13979 | 1:400 | IHC |
| Anti-CCSP | Santa Cruz Biotechnology, Dallas, TX, USA | sc-9772 | 1:1000 | IHC |
| Anti-CD4 | Santa Cruz Biotechnology, Dallas, TX, USA | sc-13573 | 1:50 | IHC |
| Anti-CD8 | Thermo Fisher Scientific, Waltham, MA, USA | MA1-70041 | 1:50 | IHC |
| Anti-mouse IgG Biotinylated | Vector Laboratories, Burlingame, CA, USA | BMK-2202 | 1:200 | IHC |
| Goat anti-rat IgG Biotinylated | Vector Laboratories, Burlingame, CA, USA | BA9401 | 1:100 | IHC |
| Goat anti-rabbit IgG Biotinylated | Dako, Carpinteria, CA, USA | E0432 | 1:200 | IHC |
| Rabbit anti-goat IgG Biotinylated | Santa Cruz Biotechnology, Dallas, TX, USA | sc-2774 | 1:200 | IHC |
| Anti-PDLIM2 (mouse) | Everest Biotech, Ramona, CA, USA | EB11878 | 1:400 | IHC |
| Anti-PDLIM2 (human) | Sigma-Aldrich, St. Louis, MO, USA | HPA003880 | 1:250 (IHC) & 1:1000 (WB) | IHC & WB |
| Anti-STAT3 | Cell Signaling Technology, Danvers, MA, USA | 4904 | 1:1000 | IHC & WB |
| Anti-RelA | Cell Signaling Technology, Danvers, MA, USA | 8242 | 1:500 (IHC) & 1:1000 (WB) | IHC & WB |
| Anti-c-Myc | Santa Cruz Biotechnology, Dallas, TX, USA | sc-40 | 1:1000 | WB |
| Anti-Hsp90 | Santa Cruz Biotechnology, Dallas, TX, USA | sc-13119 | 1:1000 | WB |
| Anti-LaminB | Santa Cruz Biotechnology, Dallas, TX, USA | sc-6217 | 1:1000 | WB |
| Anti-Sp1 | Santa Cruz Biotechnology, Dallas, TX, USA | sc-59 | 1:1000 | WB |
| Goat anti-mouse IgG-HRP | Santa Cruz Biotechnology, Dallas, TX, USA | sc-2055 | 1:5000 | WB |
| Goat anti-rabbit IgG-HRP | Santa Cruz Biotechnology, Dallas, TX, USA | sc-2054 | 1:5000 | WB |
| Anti-HDAC1 | Millipore, Burlington, MA, USA | 17-10199 | 2 µg (ChIP) & 1:1000 (WB) | ChIP & WB |
| Anti-phospho HDAC1 (Ser421/Ser423) | Millipore, Burlington, MA, USA | 07-1575 | 1:100 (ChIP) & 1:1000 (WB) | ChIP & WB |
| Anti-acetyl-Histone H3 (Lys14) | Millipore, Burlington, MA, USA | 07-353 | 1:50 | ChIP |
| Normal mouse IgG | Millipore, Burlington, MA, USA | 12-371B | 2 µg | ChIP |
| Normal rabbit IgG | Millipore, Burlington, MA, USA | PP64B | 2 µg | ChIP |
| Anti-Pol II | Cell Signaling Technology, Danvers, MA, USA | 2629 | 1:50 | ChIP |
| Anti-IFNγ FITC | eBioscience, San Diego, CA, USA | 11-7311-82 | 1 µl per $10^6$ cell | FACS |
| Anti-GranzB FITC | eBioscience, San Diego, CA, USA | 11-8898-82 | 0.25 µl per $10^6$ cell | FACS |
| Anti-CD3 PE | eBioscience, San Diego, CA, USA | 12-0031-83 | 2.5 µl per $10^6$ cell | FACS |
| Anti-CD4 PE-Cy7 | eBioscience, San Diego, CA, USA | 25-0042-82 | 1.25 µl per $10^6$ cell | FACS |
| Anti-CD8a APC | eBioscience, San Diego, CA, USA | 17-0081-83 | 0.625 µl per $10^6$ cell | FACS |
| Anti-CD11c FITC | eBioscience, San Diego, CA, USA | 11-0114-82 | 0.5 µl per $10^6$ cell | FACS |
| Anti-CD11b PE | eBioscience, San Diego, CA, USA | 12-0112-82 | 0.625 µl per $10^6$ cell | FACS |
| Anti-PD-L1 PE-Cy7 | eBioscience, San Diego, CA, USA | 25-5982-82 | 0.625 µl per $10^6$ cell | FACS |
| Anti-F4/80 APC | eBioscience, San Diego, CA, USA | 17-4801-82 | 2 µl per $10^6$ cell | FACS |
| Anti-PD-1 | BioXcell, West Lebanon, NH, USA | BE0273 | 200 µg per mouse | *in vivo* blockade |

# Supplementary Table 8. Primers Used

| Gene | Species | Accession number | Forward (5' to 3') | Reverse (5' to 3') | Usage |
|---|---|---|---|---|---|
| *gapdh* | human | NM_002046.3 | CCGAGCCACATCGCTCAGACAC | GTGACCAGGCGCCCAATACGAC | RT-PCR |
| *pdlim2* | human | NM_021630.5 | GTATGGCGTTGACGGTGGATGTG | GGAGGTCAGCGTCCTTGGCTTT | RT-PCR |
| *hdac1* | human | NM_004964.2 | ACTACGACGGGGATGTTGGA | CAGCATTGGCTTTGTGAGGG | RT-PCR |
| *bcl-2* | human | NM_000633.2 | ATGTGTGTGGAGAGCGTCAACC | TGAGCAGAGTCTTCAGAGACAGCC | RT-PCR |
| *mmp9* | human | NM_004994.2 | GCCTTTGGACACGCACGACG | AGCCCACTTGGTCCACCTGGTT | RT-PCR |
| *abcb1 (mdr1)* | human | NM_000927.4 | GCGAGGTCGGAATGGATCTT | GCCAAAGTTCCCACCACCAT | RT-PCR |
| *bcl2l1 (bcl-xl)* | human | NM_138578.1 | GAATGACCACCTAGAGCCTTGG | TGTTCCCATAGAGTTCCACAAAAG | RT-PCR |
| *birc5 (survivin)* | human | NM_001168.2 | TGACGACCCCATAGAGGAACA | CGCACTTTCTCCGCAGTTTC | RT-PCR |
| *ccnd1 (cyclin D1)* | human | NM_053056.2 | GCTGCGAAGTGGAAACCATC | GCACTTCTGTTCCTCGCAGA | RT-PCR |
| *cd274 (pd-l1)* | human | NM_014143.3 | TGGCATTTGCTGAACGCATTT | AGTGCAGCCAGGTCTAATTGT | RT-PCR |
| *cdh2 (n-cadherin)* | human | NM_001792.3 | GGCTTCTGGTGAAATCGCAT | GCAGGCTCACTGCTCTCATA | RT-PCR |
| *β-actin* | mouse | NM_007393.3 | ACCCGCGAGCACAGCTTCTTTG | CTTTGCACATGCCGGAGCCGTTG | RT-PCR |
| *gapdh* | mouse | NM_008084.2 | AGTGCCAGCCTCGTCCCGTA | CAGGCGCCCAATACGGCCAA | RT-PCR |
| *pdlim2* | mouse | NM_145978.2 | GAGAACATGCTACACGCGGA | GGAGCCCTGGAATCTGGTTG | RT-PCR |
| *h2-k1* | mouse | NM_001001892.2 | AGGCTGGTGAAGCAGAGAGA | ATGTCAGCAGGGTAGAAGCC | RT-PCR |
| *twist2* | mouse | NM_007855.2 | CTACCAGGTTCTCCAGAGCG | TTGTCCAGGTGCCGAAAGTC | RT-PCR |
| *sftpc (sp-c)* | mouse | NM_011359.2 | AAAGAGGTCCTGATGGAGAGTCCAC | GCTCCTGGGACCTGCCGAGTA | RT-PCR |
| *bcl2l1 (bcl-xl)* | mouse | NM_009743.4 | AGATTCAGCACGAGCAGTCA | GGGCTCAACCAGTCCATTGT | RT-PCR |
| *ccnd1 (cyclin D1)* | mouse | NM_007631.2 | CAAAATGCCAGAGGCGGATG | CATGGAGGGTGGGTTGGAAA | RT-PCR |
| *cd274 (pd-l1)* | mouse | NM_021893.3 | CCTGCTGTCACTTGCTACGG | CACTAACGCAAGCAGGTCCA | RT-PCR |
| *cdh2 (n-cadherin)* | mouse | NM_007664.4 | CCTTGCTTCAGGCGTCTGTG | CTTGAAATCTGCTGGCTCGC | RT-PCR |
| *hdac1* | human | NM_004964.2 | CTGTGAGGAAGAGTTCGCCGATGCTGAAGAGGAGGGAGAG | CTCTCCCTCCTCTTCAGCATCGGCGAACTCTTCCTCACAG | Mutation |
| *hdac1* | human | NM_004964.2 | TGCTGCTCAACTATGGTCTCTATTCAAGAGATAGAGACCATAGTTGAGCAGCTTTTTTC | TCGAGAAAAAAGCTGCTCAACTATGGTCTCTATCTCTTGAATAGAGACCATAGTTGAGCAGCA | ShRNA |
| *hdac1* | human | NM_004964.2 | GGgcggccgcAAGATGGCGCAGACGCAGG | AGggatccTCAGGCCAACTTGACCTCCTC | Cloning |
| *pdlim2* | human | Gene ID: 64236 | GAAGTGAAACCGGGCTGAGG | GCCAAAGGGAGAAGGAGAGG | ChIP PCR |
| *pdlim2* | human | Gene ID: 64236 | AGAGGAGTTTATATATATTTAGG | TACCTAACAACCCTCTCTCC | Bisulfite PCR |
