# Supplementary figures and images for: Causative role of PDLIM2 epigenetic repression in lung cancer and therapeutic resistance
Source: Nat Commun. 2019 Nov 22;10:5324. doi: 10.1038/s41467-019-13331-x (PMC6876573; doi:10.1038/s41467-019-13331-x)

Fig. 1d

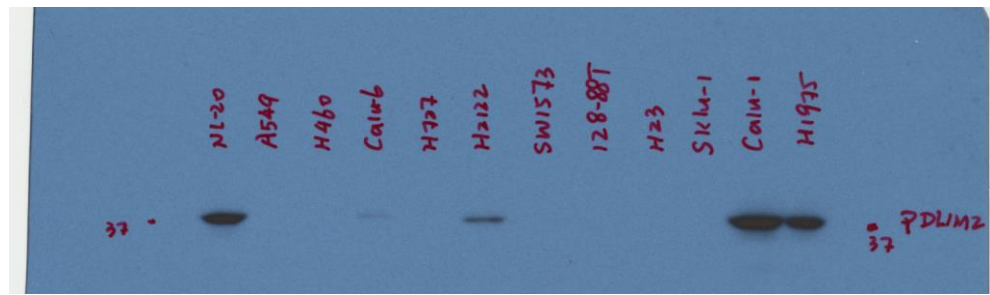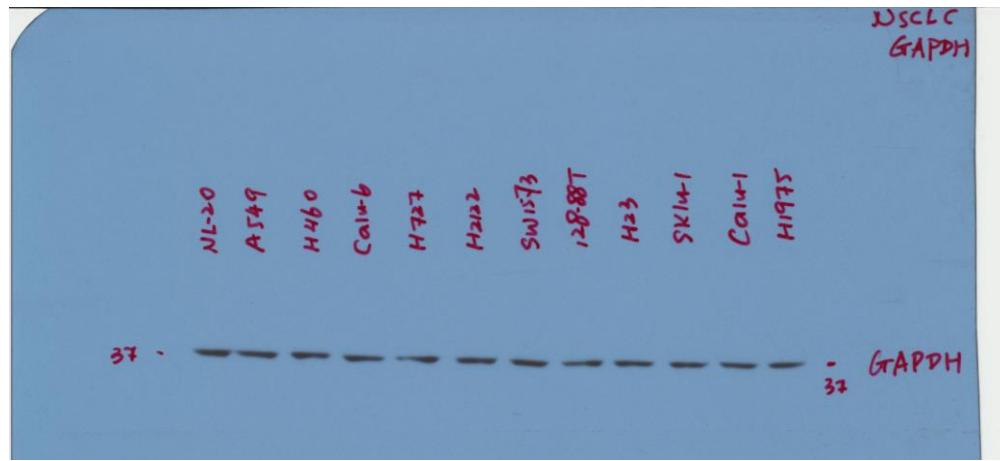

Fig. 2k

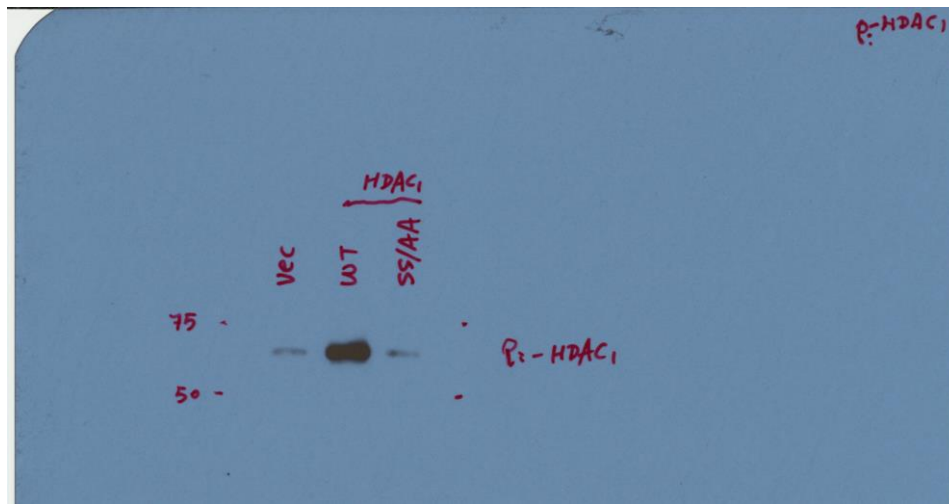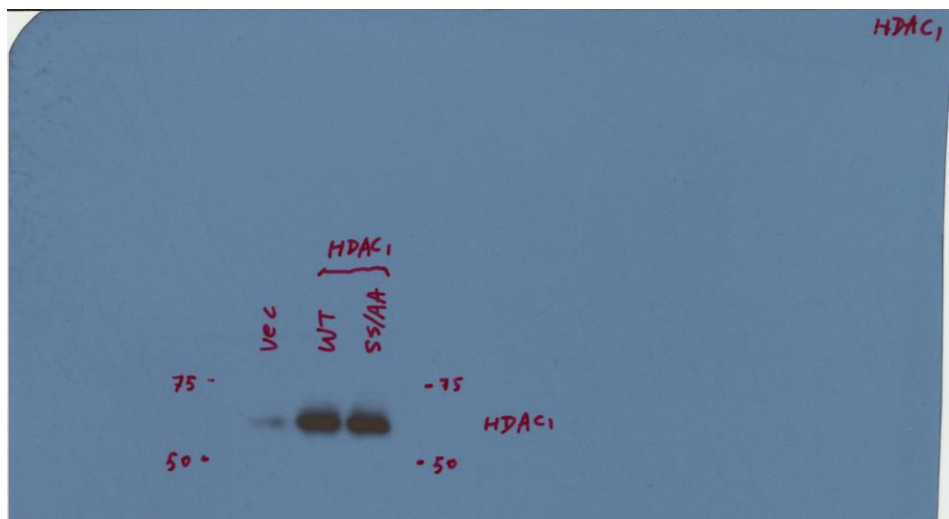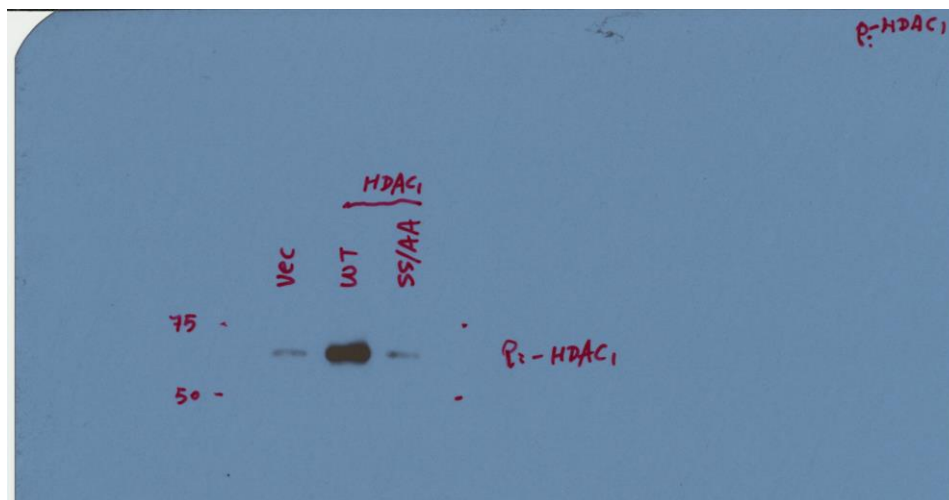

Fig. 6b

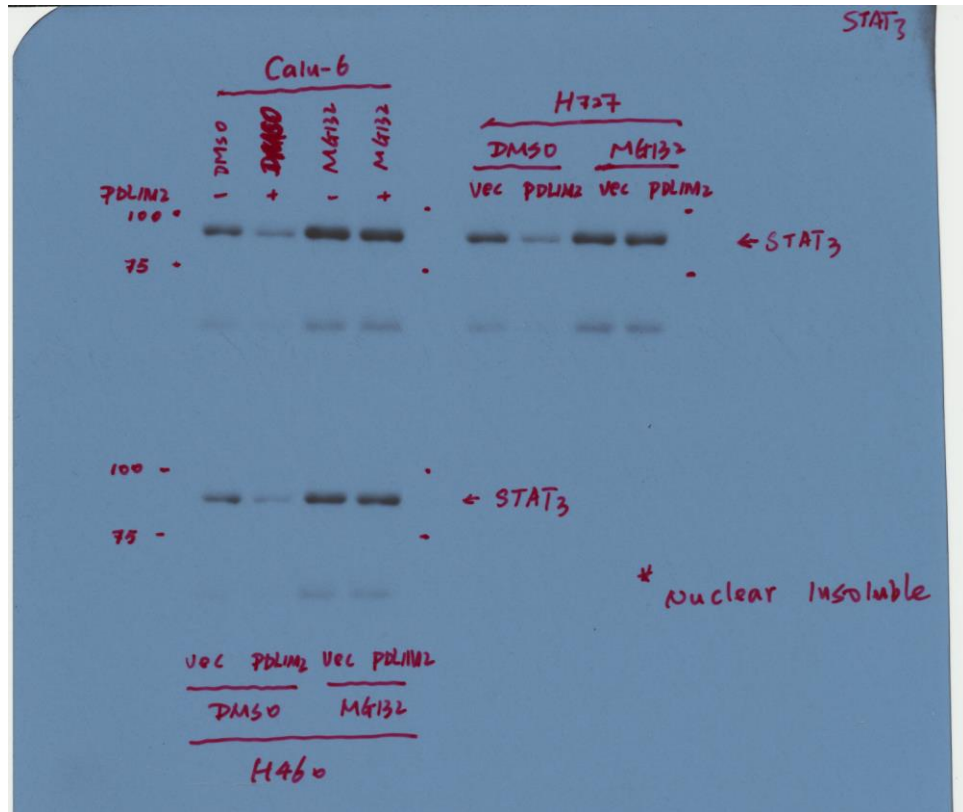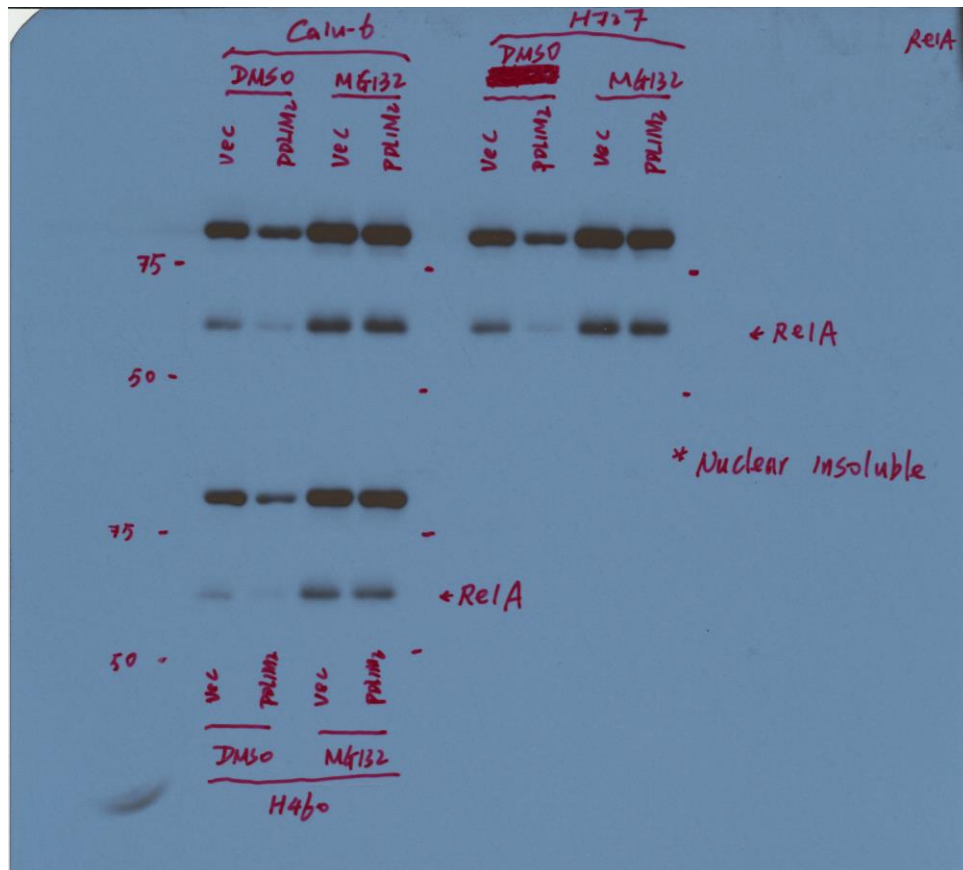

Fig. 6b

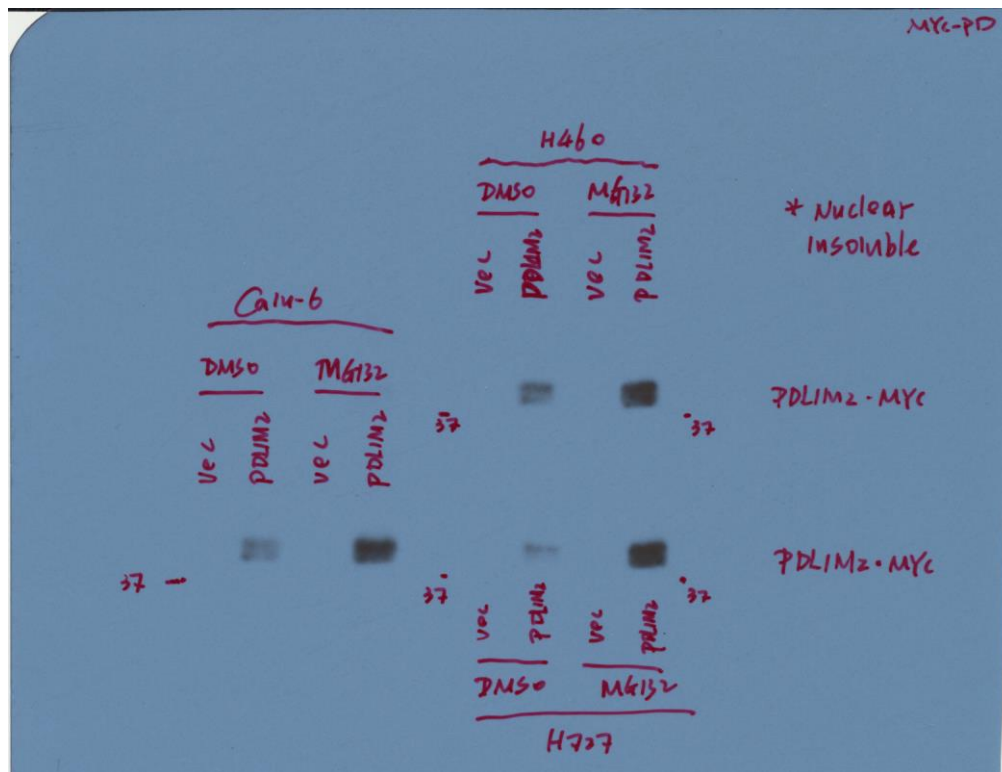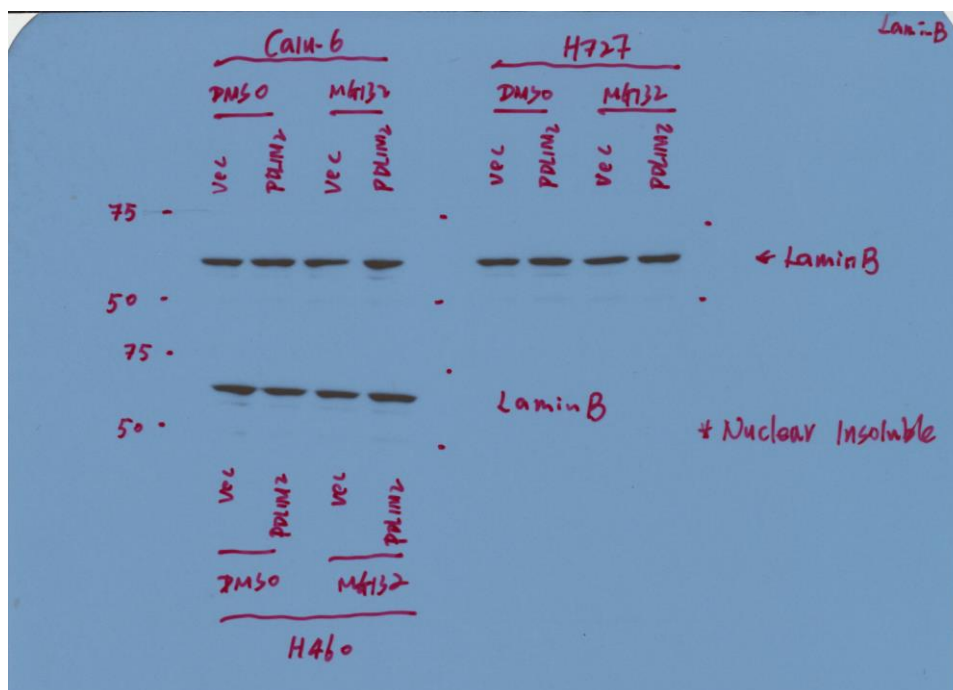

Fig. 6g

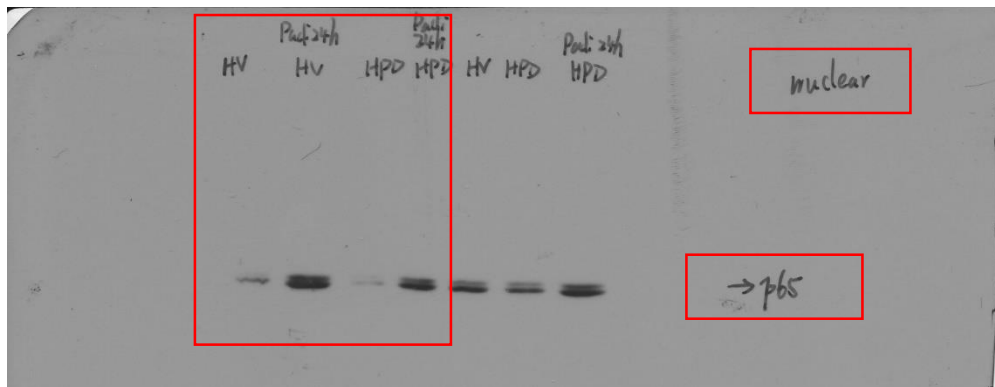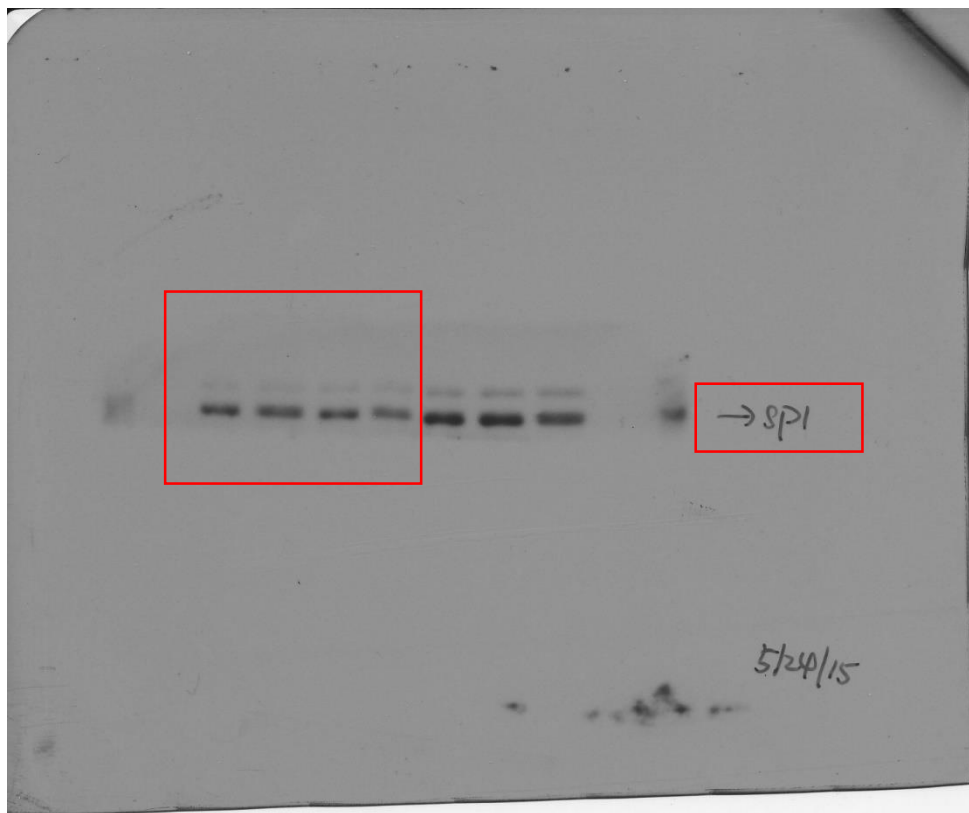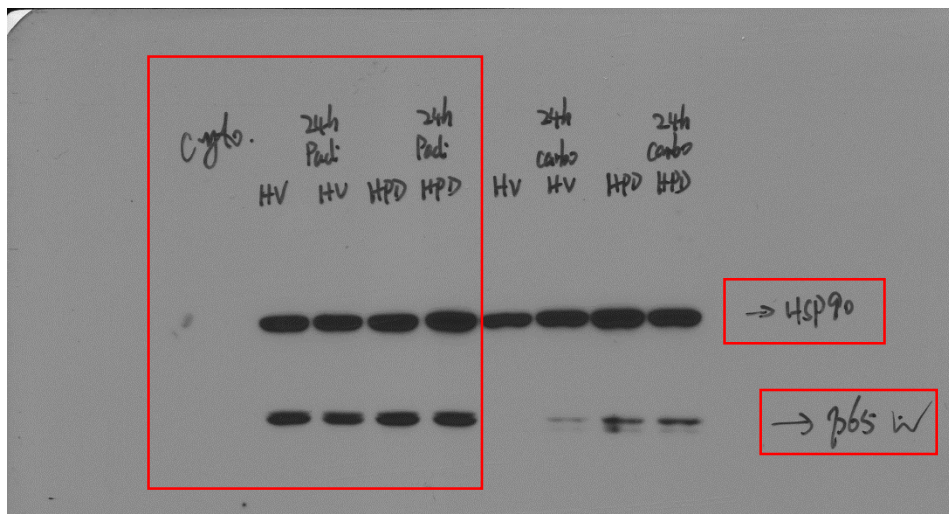

Supplement: Supplementary file 3 — Source Data [file 41467_2019_13331_MOESM3_ESM.pdf]
